# Super-elastic and fatigue resistant carbon material with lamellar multi-arch microstructure

**DOI:** 10.1038/ncomms12920

**Published:** 2016-09-27

**Authors:** Huai-Ling Gao, Yin-Bo Zhu, Li-Bo Mao, Feng-Chao Wang, Xi-Sheng Luo, Yang-Yi Liu, Yang Lu, Zhao Pan, Jin Ge, Wei Shen, Ya-Rong Zheng, Liang Xu, Lin-Jun Wang, Wei-Hong Xu, Heng-An Wu, Shu-Hong Yu

**Affiliations:** 1 Division of Nanomaterials & Chemistry, Hefei National Laboratory for Physical Sciences at the Microscale, Collaborative Innovation Center of Suzhou Nano Science and Technology, Department of Chemistry, CAS Center for Excellence in Nanoscience, Hefei Science Center of CAS, University of Science and Technology of China, Hefei 230026, China; 2 CAS Key Laboratory of Mechanical Behavior and Design of Materials, Department of Modern Mechanics, University of Science and Technology of China, Hefei, Anhui 230027, China; 3 Nano-Materials and Environmental Detection Laboratory, Hefei Institute of Intelligent Machines, Chinese Academy of Sciences, Hefei, Anhui 230031, China

## Abstract

Low-density compressible materials enable various applications but are often hindered by structure-derived fatigue failure, weak elasticity with slow recovery speed and large energy dissipation. Here we demonstrate a carbon material with microstructure-derived super-elasticity and high fatigue resistance achieved by designing a hierarchical lamellar architecture composed of thousands of microscale arches that serve as elastic units. The obtained monolithic carbon material can rebound a steel ball in spring-like fashion with fast recovery speed (∼580 mm s^−1^), and demonstrates complete recovery and small energy dissipation (∼0.2) in each compress-release cycle, even under 90% strain. Particularly, the material can maintain structural integrity after more than 10^6^ cycles at 20% strain and 2.5 × 10^5^ cycles at 50% strain. This structural material, although constructed using an intrinsically brittle carbon constituent, is simultaneously super-elastic, highly compressible and fatigue resistant to a degree even greater than that of previously reported compressible foams mainly made from more robust constituents.

## Abstract (web summary)

Low-density compressible materials often suffer from fatigue-induced failure or limited elasticity. Here, the authors create a hierarchical multi-arch carbon material that achieves high compressibility, superior elasticity and fatigue resistance simultaneously, inspired by properties of arches in daily life.

## Graphical abstract

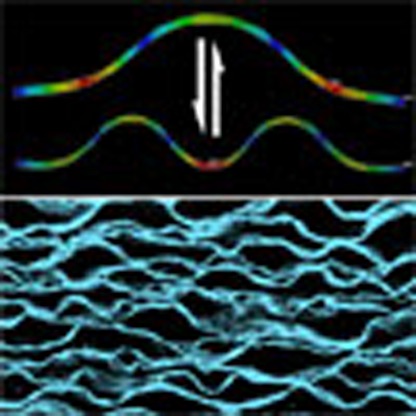

Low-density compressible materials with high elasticity and fatigue resistant ability have demonstrated promise for applications such as mechanical cushioning, energy damping and flexible devices^1^,^2^,^3^,^4^,^5^,^6^,^7^,^8^,^9^,^10^,^11^,^12^,^13^. Compressibility, elasticity and fatigue resistance are three main factors that determine the performance and applications of these materials^4^,^5^,^9^. Various strategies have been exploited to improve these properties, focusing primarily on using specific cellular microstructure^2^,^6^,^7^,^8^,^14^ and flexible but robust solid constituents^3^,^9^,^15^,^16^,^17^. Within this research area, graphene has become one of the most popular constituents^7^,^8^,^9^,^18^,^19^,^20^ due to its intrinsic physical and chemical properties^21^,^22^,^23^,^24^,^25^.

While high compressibility has been demonstrated^9^,^14^,^15^,^18^,^26^,^27^,^28^, achieving superior elasticity with fast recovery rate and small energy dissipation, as well as high fatigue resistant ability under high strain cyclic compression has remained challenging. These properties are, in general, mutually exclusive and hard to attain simultaneously. Specifically, permanent failure by buckling and/or fracture is typical for traditional open-cell foams when they are under high strain cyclic compression, leading to large energy dissipation, plastic deformation and reduced strength under compression^1^,^2^,^3^,^4^. Rational design of microstructure is an attractive research avenue for alleviating these problems.

Considering examples from daily life, we found some macrostructures that can provide a conceptual basis for microstructural solutions. For example, leaf springs, the basis for one of the oldest forms of arch-shaped spring-type suspension systems widely used in vehicles, help to support the axle and absorb shock. Another prototypical example is the arch of the foot, which also acts as an elastic spring-type cushioning system to minimize the risk of musculoskeletal wear or damage and facilitate walking, running and jumping^29^. These two arch-shaped macrostructures are notable for combining elasticity and fatigue resistance.

Herein, inspired by macroscale arch-shaped elastic structures, we create a super-elastic, lightweight monolithic carbon–graphene (C–G) composite composed of numerous multi-arch microscale structures arranged into parallel stacks. The unique hierarchical architecture is achieved by applying a bidirectional freezing process to obtain a chitosan–graphene oxide (CS–GO) scaffold composed of parallel flat lamellas with long-range alignment and by subsequent annealing to crumple the flat lamellas into a waved multi-arch morphology. Derived from the designed unique lamellar multi-arch microstructure, the obtained monolithic carbon material exhibits spring-like super-elasticity, high compressibility and superior fatigue resistance simultaneously, which is distinct from previously reported compressible foams.

## Results

### Fabrication of super-elastic C–G monolith

**Figure 1:**
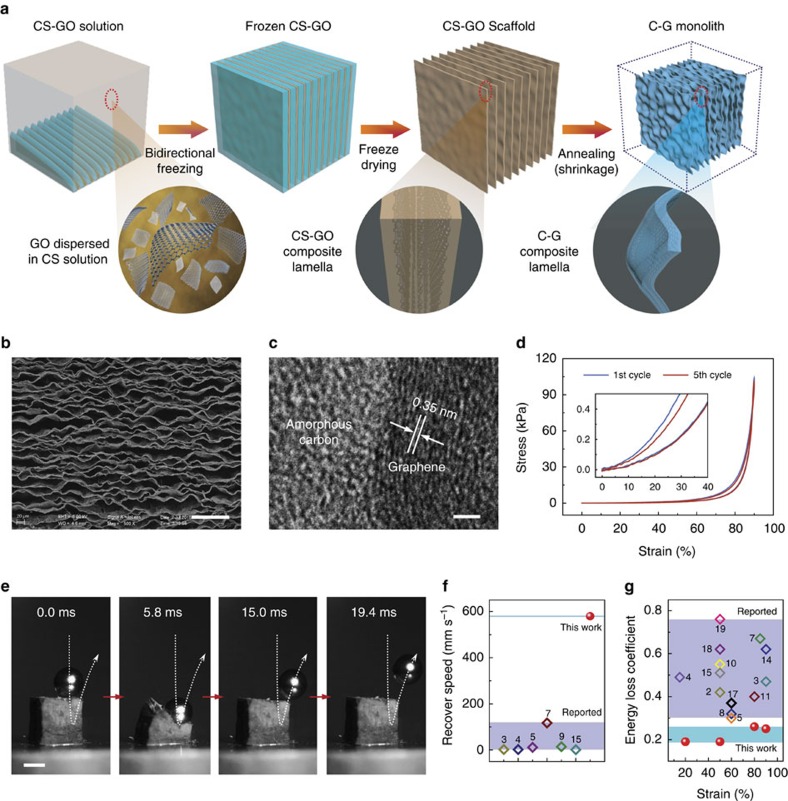
Structure design and compressive elasticity. (**a**) Schematic illustration of the fabrication of carbon–graphene (C–G) monolith. (**b**) SEM image (top view) of C–G monolith shows the lamellar multi-arch microstructure with long-range alignment. Scale bar, 100 μm. (**c**) High resolution transmission electron microscopy image shows that the lamella is composed of amorphous carbon and graphene composite. Scale bar, 2 nm. (**d**) Stress–strain curves of C–G monolith under high strain compression. (**e**) Real-time images from high-speed camera showing that C–G monolith can rebound a steel ball at large speed, in a springlike fashion. Scale bar, 4 mm. (**f**,**g**) Recovery speed (**f**) and energy loss coefficient (**g**) of C–G monolith and other previously reported materials. Numbers in the charts represent relevant references.

To obtain the desired lamellar structure, first we developed an optimized facile bidirectional freezing method^30^ to prepare lamellar CS–GO scaffold with homogeneous mixture of CS and GO suspension as raw material (Fig. 1a; Supplementary Figs 1–3; Supplementary Movie 1). Through this procedure, CS–GO scaffold composed of parallel, aligned and thin lamellas was readily obtained (Supplementary Fig. 4). The lamellar CS–GO scaffold was then annealed under inert gas to induce the formation of multi-arch microstructures (Fig. 1a). In this annealing process, local volume decrease is much more pronounced in the CS matrix than in the embedded GO sheets due to greater relative material loss under heating in the CS matrix (Supplementary Fig. 5). Thus, the relative flat thin lamellas were crumpled into waved multi-arch morphology due to the uneven stress distribution in the lamellas^12^ (Fig. 1b; Supplementary Fig. 4). Simultaneously under annealing, GO sheets embedded in the CS matrix are reduced and CS is carbonized into amorphous carbon^31^,^32^ (Fig. 1c; Supplementary Fig. 6), welding the reduced GO sheets together into thin C–G lamellas (Supplementary Fig. 4f). The total carbon content in the ultimate C–G monolith was measured to be ∼75.8 wt% (Supplementary Fig. 7). Considering the initial CS content (78.7 wt%) in CS–GO scaffold (Supplementary Fig. 7) and thermogravimetric analysis results of pure CS and GO (Supplementary Fig. 5b), we believe the main carbon constituent in the ultimate C–G monolith to be amorphous carbon from the carbonized CS matrix. For a typical C–G monolith with density of 14.1 mg cm^−3^, the Brunauer–Emmett–Teller (BET) specific surface area was measured to be a relatively low value (51.36 m^2^ g^−1^), corresponding to a typical macroporous material. Notably, the obtained C–G monoliths exhibit anisotropic electrical conductivity (with ∼6.7 S m^−1^ along the lamella direction and ∼1.4 S m^−1^ perpendicular to the lamella direction, Supplementary Fig. 8), which can be attributed to the anisotropic lamellar structure.

As we expected, CS–GO scaffold with relatively flat lamellas displayed weak elasticity and did not completely spring back to its original height (Supplementary Fig. 9a). On the other hand, it changed into a super-elastic C–G monolith after the flat lamellas crumpled into multi-arch microstructures (Supplementary Fig. 9b). In contrast, monoliths made from pure GO and CS through the same manufacturing process suffered from serious plastic deformation and from brittle collapse, respectively (Supplementary Fig. 9c–f). Remarkably, as shown in Fig. 1d and Supplementary Movie 2, the C–G monolith can completely recover to its original height upon 90% compression strain without yielding or plastic deformation, and no lateral extension happens during the compression process. Movie from a high-speed camera shows that a typical C–G monolith can rebound a steel ball (0.87 g, 100 times heavier than itself) with a fast recovery speed (∼580 mm s^−1^) (Fig. 1e; Supplementary Movie 3), which is much faster than previously reported results (Fig. 1f; Supplementary Table 1), revealing elastic performance with instantaneous recovery similar to that of a spring.

Notably, the typical crescent-shaped stress–strain curves of C–G monolith (Fig. 1d) are distinct from that of conventional open-cell foams which show three characteristic deformation regions: an initial linear elastic region, relating to bending of the cell walls; a relatively flat plateau region, relating to buckling of cell walls or yielding of the foams with plastic deformation; and a final region of increasing stress, relating to densification of cells^3^,^5^,^7^,^8^,^18^. Furthermore, the hysteresis loop in a typical stress–strain curve of the C–G monolith was found to be very narrow in comparison to previously reported results for open-cell foams (Fig. 1d). The energy dissipation in each cycle was calculated to be only about 0.2 (even with compression strains up to 90%), and this loss is much lower than that in previously reported works (Fig. 1d,g; Supplementary Table 1)^2^,^3^,^5^,^33^, indicating much lower energy dissipation under the compress-release cycles. The characteristic shape and hysteresis loop of the stress–strain curves suggest that microstructural buckling or damage in the compression process of the C–G monolith is far more limited than in other materials, differing noticeably from for example, traditional viscoelastic materials^4^,^33^,^34^,^35^ and resembling more an elastic rubber^6^. More evidence for enhanced elastic behaviour was provided by a static compression test, which revealed that the elastic strength could be maintained at a constant value and essentially no viscoelastic-like stress-relaxation behaviour was observed when the sample was compressed and held at a certain strain (50%) level^4^ (Supplementary Fig. 10).

### Mechanistic investigation of the mechanical properties

**Figure 2:**
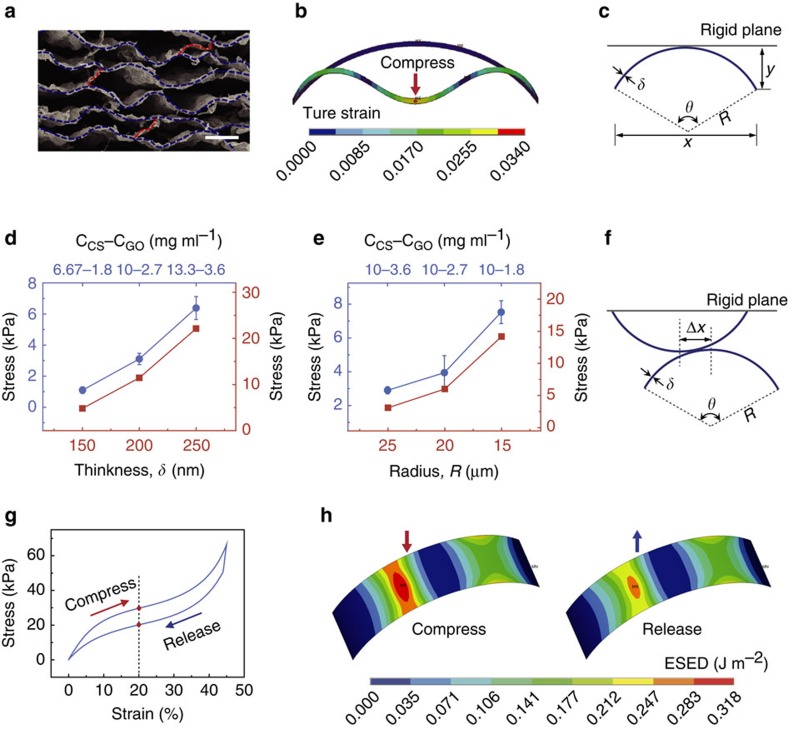
Mechanical analysis and simulations. (**a**) Microstructure of C–G monolith, showing randomly distributed bridge ligaments (marked in red dotted lines) linking adjacent lamellas. Scale bar, 20 μm. (**b**) The true material strain (von Mises total strain) profiles of cylindrical shell under large geometry deformation. (**c**) Schematic cross-section view of cylindrical shell mode under compression by a rigid plane. (**d**) Compression stresses of bulk C–G monoliths with different lamella thickness (blue) and single cylindrical thin-shell mode with different shell thickness (red). (**e**) Compression stresses of bulk C–G monoliths with different shrinkage (blue) and single cylindrical thin-shell mode with different radius (red). C_CS_–C_GO_ represents the concentration of CS and GO in the initial CS–GO composite suspensions for fabricating the C–G monoliths. (**f**) Schematic diagram of two opposite cylindrical shells with offset distance Δ*x* compressed by a rigid plane. (**g**) Simulated stress–strain curve based on two opposite cylindrical shells with offset distance Δ*x*=0.4*R* in a compress-release cycle. (**h**) The elastic strain energy density profiles of cylindrical shell when the strain equal to 20% in compression and release processes (**g**), respectively. All error bars represent the s.d. of at least six replicate measurements.

The highly compressible and super-elastic behaviour of the C–G monoliths can be attributed to the characteristic lamellar multi-arch microstructure, which was systematically investigated by both mechanical simulations and experiments. Figure 2a and Supplementary Fig. 11 show that adjacent lamellas are linked with each other through randomly distributed bridge ligaments. These ligaments should help restrict lateral extension of C–G monolith when it undergoes compression perpendicular to its lamella direction. Therefore, an arch-shaped cylindrical thin-shell with simply supported boundary can be applied here as the simplified structural element of C–G monolith to characterize its structural and mechanical features (Supplementary Fig. 12a).

Generally, thin-shell structures easily undergo large out-of-plane deformation yet have small in-plane strain. Mechanical simulations using the finite element method revealed that this kind of arch-shaped cylindrical thin-shell model can sustain large geometric deformation without yielding because of its small material strain, and it can also spring back to its original shape immediately (Fig. 2b; Supplementary Fig. 12b). Furthermore, due to the consistence of the preferred lamella direction across the whole C–G monolith, all arch microstructures exhibit preferred alignment along the lamella direction in a staggered stacking manner (Supplementary Fig. 4h). Therefore, in direction perpendicular to the lamellas, C–G monolith can undergo large geometric deformation without structural collapse due to large out-of-plane deformation of its waved lamellas. Meanwhile, it is relatively easy to damage for the other two lateral directions due to buckling failure and fracture of the lamellas (Supplementary Fig. 13). The elastic behaviour of this simplified cylindrical thin-shell model was investigated by compressing it with a rigid plane (Fig. 2c). The simulated stress–strain curves in compress-release process for different geometry parameters (radius *R*, thickness *δ*) reveal typical of nonlinear elasticity (Supplementary Fig. 12c,d). It was found that thicker (bigger *δ*) and smaller (smaller *R*) cylindrical shells showed higher elastic strength (Fig. 2d,e).

Experimentally, we prepared a series of C–G monoliths with different geometric parameters characterizing the arch microstructure through adjusting the initial content of GO and CS. Solid content mainly determines the thickness *δ* of the arch-shell and the weight ratio of GO to CS mainly determines the radius *R* of the arch microstructure (Fig. 2d,e; see Supplementary Figs 14–18). Compression tests of the C–G monoliths exhibit the expected nonlinear elastic features (Supplementary Fig. 14g–i). Moreover, C–G monoliths with thicker lamella (Fig. 2d; Supplementary Fig. 14g) and smaller arch microstructure (Fig. 2e; Supplementary Fig. 14h) exhibit higher elastic strength, displaying consistency with the simulated results of the cylindrical thin-shell mode. The mechanical behaviour of the bulk C–G monoliths can be regarded as a result of the collective behaviour of thousands of arch-shaped cylindrical shells.

Note that narrow hysteresis loops were observed in the stress–strain curves for experiments (Fig. 1d) but not in the simulations of simplified cylindrical thin-shell model (Supplementary Fig. 12c,d). In general, except for buckling or cracking of microstructures, friction between cellular walls or struts is the other main factor related to hysteresis loops^3^,^7^,^8^. As shown in Fig. 1b, opposite, staggered micro-arches with random offset distances are common in the C–G monolith, and there are also many little bulbs and wrinkles within the lamellas, making their surfaces rough (Supplementary Fig. 17). Thus, we were convinced that sliding friction between the opposite arch-shells should happen in cyclic compression process of the C–G monolith. Mechanical simulation using two opposite cylindrical shells with different offset distance Δ*x* was then applied to investigate the contribution of sliding friction to the energy dissipation in compress-release process of C–G monolith (Fig. 2f). The simulated results demonstrate that there is no hysteresis loop in the stress–strain curve for small Δ*x* (Supplementary Fig. 19a) because no relative sliding happened. Only when Δ*x* exceeds a certain value (Fig. 2g; Supplementary Fig. 19b), the two cylindrical shells begin to slide past each other, resulting in typical hysteresis loops due to the energy dissipation induced by sliding friction (Supplementary Fig. 19c–f). Elastic strain energy density profiles of a cylindrical shell at certain strain (20%) were extracted to illustrate the energy loss in the compress-release cycle (Fig. 2h). The simulated results together with the above-mentioned experimental results certify that the narrow hysteresis loops observed in the experiments should be mainly caused by sliding friction among the arch-shells in the compress-release process of C–G monolith.

### Structural comparison with controls

**Figure 3:**
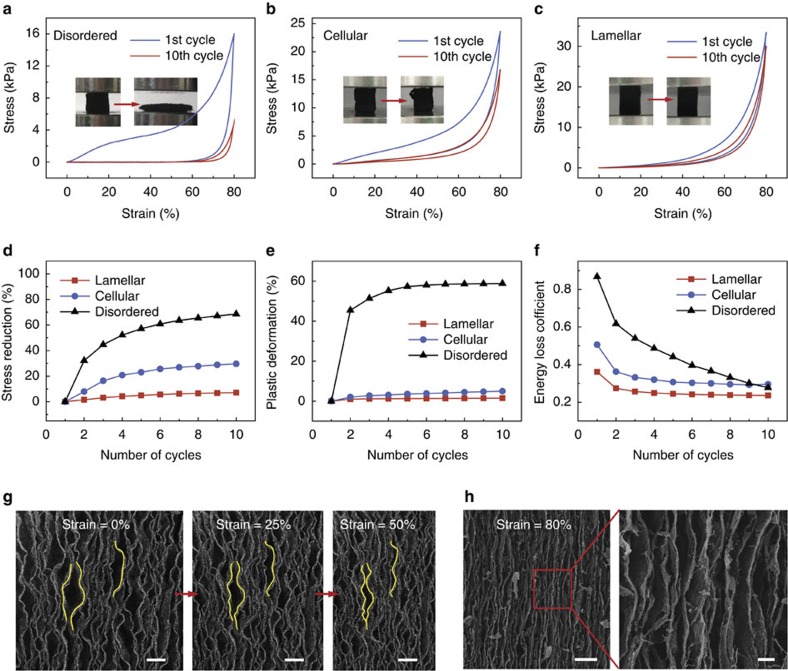
Structural performance against controls. (**a**–**c**) Stress–strain curves of the three kinds of C–G monoliths under cyclic compression and insert photos show the corresponding samples. (**d**–**f**) Changes of maximum stress (**d**), plastic deformation (**e**) and energy loss coefficient (**f**) of the three kinds of C–G monoliths during the first 10 compression cycles at the maximum strain of 80%, respectively. (**g**) *In situ* SEM observation of C–G monolith at the maximum strain of 50%. Scale bars, 50 μm. (**h**) SEM observation of C–G monolith under compression strain of 80%. Scale bars, 50 μm on left side of panel, 10 μm on right side of panel.

To further demonstrate the critical role of the designed hierarchical architecture in allowing the observed structural properties, open-cell foams with traditional disordered porous structure (Supplementary Fig. 20a) and cellular structure (Supplementary Fig. 20b) were made as control groups using the same carbon composite constituent and annealing treatment as that of lamellar C–G monolith. Disordered C–G monolith collapsed with little resistance upon compression and displayed large energy dissipation in the first compression cycle (Fig. 3a,d–f), indicating that the carbon composite constituent for constructing these C–G monoliths is actually very brittle. As the scanning electron microscope (SEM) observation showing in Supplementary Fig. 20c, many fractures of the cellular walls or struts in the disordered C–G monolith can be seen at only 50% compression strain. In the case of the cellular C–G monolith, while it also consists of thin-shell arch microstructures (waved cellular walls), these arch-shells are not preferrentially aligned a single direction (Supplementary Fig. 20b). Therefore, these arch-shells are expected to suffer from folding, buckling or structural collapse when under large strain compression perpendicular to its cellular channels (Supplementary Fig. 20d), which ultimately result in stress reduction, plastic deformation and energy dissipation of cellular C–G monolith in cyclic compression (Fig. 3b,d–f). In contrast, the lamellar C–G monoliths show relatively little reduction in strength, little permanent deformation and little energy dissipation (Fig. 3c–f).

*In situ* SEM observation reveals that no buckling failure or structural collapse of the lamellas occurs in the compress-release cycles (Fig. 3g; Supplementary Movie 4), and even upon 80% compressive deformation, the lamellas are still weaved instead of flat or fracture (Fig. 3h). We notice that, large arch-shells generally tend to deform into several small arch-shells by large out-of-plane deformation and can instantly spring back to their original shape (Fig. 3g; Supplementary Movies 4 and 5) when the applied loads are released. These observed results are in good accordance with the simulated results in Fig. 2b and Supplementary Fig. 11b. Therefore, this specific lamellar architecture with multi-arch microstructure is particularly effective for tolerating large geometric deformation yet preventing structural damage or collapse.

Further multi-cycle compression tests of the C–G monoliths revealed addtitional effects of the geometry of the arch-shells on structural performance (Supplementary Fig. 21). For example, C–G monoliths with larger arch-shells can accommodate larger out-of-plan deformation, while those with smaller arch-shells are more likely to suffer from structural damage (Supplementary Figs 15 and 21a–c). Moreover, C–G monoliths with relatively flat arch-shells often undergo a certain extent of plastic deformation during the first compression cycle, and if the lamellas had more defects (holes on the lamellas) (Supplementary Figs 15b, 16b), the relevant C–G monolith would dissipate much more energy in the compression cycles (Supplementary Fig. 21d–f).

### Fatigue resistance testing

**Figure 4:**
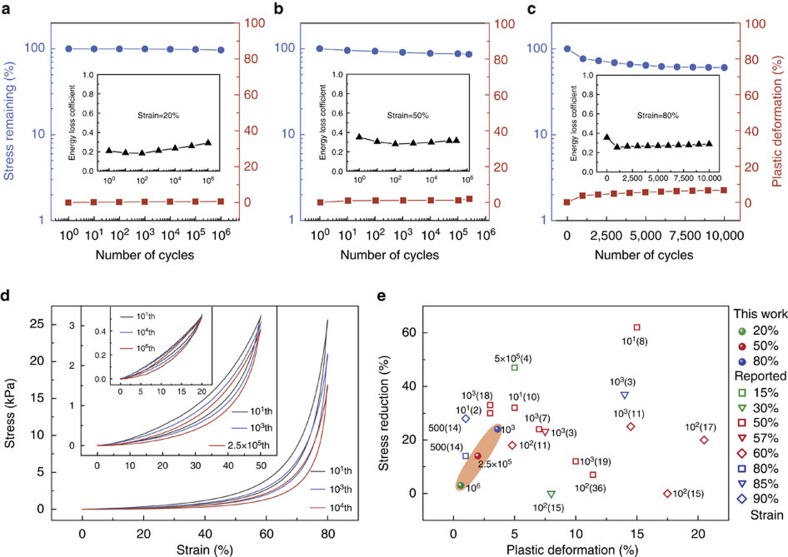
Fatigue resistance. (**a**–**c**) Elastic strength, plastic deformation and energy loss coefficient during 1 × 10^6^ cycles at 20% strain (**a**), 2.5 × 10^5^ cycles at 50% strain (**b**) and 1 × 10^4^ cycles at 80% strain (**c**). (**d**) Stress–strain curves of C–G monoliths at 20% strain for 1 × 10^6^ cycles, at 50% strain for 2.5 × 10^5^ cycles and at 80% strain for 1 × 10^4^ cycles. (**e**) Ashby chart plotting stress reduction versus plastic deformation for C–G monolith (10–3.6) and other previously reported materials. Numbers of compression cycle are marked beside the corresponding dots in the chart. Numbers in paranthesis represent relevant references. Per cent values shown to the right of the chart list the relevant compression strain for each finding.

The lamellar C–G monolith (10–3.6) was further tested at different compress-release cycles with different compression strains to test its fatigue resistant ability. Remarkably, Figure 4a,d shows negligible change in the maximal stress (∼3%) and volume deformation (0.6%) at a strain level of 20% even after 1 × 10^6^ compression cycles. Furthermore, after being compressed for another 2.5 × 10^5^ cycles at a strain level of 50%, the C–G monolith still maintained over 86% of maximum stress and suffered only 2% permanent deformation (Fig. 4b,d). These results illustrate that C–G monolith tolerate large elastic deformation without accumulating damage or undergoing structural collapse. Moreover, even under more harsh compression condition (80% strain for 10^4^ cycles), C–G monolith still retained over 60% of its maximum stress and showed only 7% reduction in height (Fig. 4c,d), with these changes occurring mainly in the earlier cycles and the structure becoming relatively stable in the subsequent cycles (Supplementary Fig. 21d,e). The minor structural damage under cyclic compression leads to small changes in the energy loss coefficient with cycling (Fig. 4a–c). It is noteworthy that C–G monolith employing intrinsically brittle carbon composite as solid constituent is even more stable than foams made from strong and robust solid constituents (for example only containing graphene or carbon nanotubes)^36^ (Fig. 4e, Supplementary Table 1).

## Discussion

In conclusion, the lamellar multi-arch design presented here overcomes the brittleness of the carbon constituent and endows the C–G monolith with high compressibility, super-elasticity and fatigue resistance simultaneously. These mechanical properties, together with low density and high electrical conductivity, imply many potential application areas, such as for flexible piezoresistive sensors^6^. It is expected that this design concept might be combined with three-dimensional (3D) printing to enable further advances in compressible structural materials. As the original inspiration for this work lies in the mechanical performance of specific macrostructures, one may reasonably speculate that many mature theories in structural mechanics for macrostructures could provide guidelines for developing microstructures to achieve unique mechanical properties.

## Methods

### CS–GO suspension preparation

Chitosan (CS, Degree of deacetylation ≥95%, Viscosity 100–200 Mpa·s, Aladdin) solution (40 mg ml^−1^) was prepared by dissolving chitosan powders in aqueous solution with 4% acetic acid. Graphene oxide (GO) was prepared by oxidizing natural graphite powders via a modified Hummers' method reported elsewhere^37^. The obtained GO were treated with ultrasonication (500 W) for 5 min (JY92-IID). Then CS and GO were mixed together at a certain weight ratio, followed by ultrasonic treatment (800 W) with an ultrasonic processor for 10 min to make sure GO sheets be sufficient dispersed in CS matrix.

### Fabrication of C–G monoliths

First, long-range lamellar CS–GO scaffolds were prepared. CS–GO suspension was placed in a cubic silicone mould placed on the surface of a steel plate (Supplementary Figs 2 and 3). One end of the steel plate was then inserted into liquid nitrogen to induce a temperature gradient on the plate surface. Thus, the formed ice nucleus would grow along horizontal direction (0.2 mm s^−1^) to form parallel ice columns with long-range alignment, and simultaneously grow along vertical direction (0.04 mm s^−1^) to form parallel ice lamellas with long-range alignment (Supplementary Fig. 2). After complete freezing, the samples were freeze dried. The obtained CS–GO scaffolds were treated in furnace in N_2_ gas with a heating rate of 2 °C min^−1^ from room temperature to 500 °C and kept at 500 °C for 1 h, then 5 °C min^−1^ from 500 °C to 800 °C and kept at 800 °C for 2 h. Disordered C–G monoliths were prepared by freezing CS–GO suspensions in −20 °C refrigerator, followed by freeze drying and annealing using same conditions as mentioned above. Cellular C–G monoliths were prepared by unidirectional freezing of the CS–GO suspensions as our previously reported method^38^, followed by freeze drying and annealing using same conditions as mentioned above. Pure AC and G lamellar monoliths were prepared by the same method as lamellar C–G monoliths.

### Sample characterization

Atomic force microscopy image of GO nanosheets was obtained from an Dimension 3100 SPM under contact mode. Size distribution and zeta potential were tested by Malvern Nano-ZS90. Fourier transform infrared spectroscopy spectra were measured on a Bruker Vector-22 fourier transform infrared spectroscopy spectrometer from 4,000 to 400 cm^–1^ at room temperature. SEM images were collected using a field emission scanning electron microanalyzer (Zeiss Supra 40) at an acceleration voltage of 5 kV. Thermogravimetric analysis was measured on a thermal analyser (SDT Q600, TA instruments, USA) with a heating rate of 2 °C min^−1^ from room temperature to 500 °C and kept for 1 h, then 5 °C min^−1^ from 500 °C to 800 °C and kept for 2 h in N_2_ gas. X-ray photoelectron spectroscopy was performed on an ESCALAB 250 (Thermo Electron Corporation). High resolution transmission electron microscopy observation was performed on a JEM-2100F transmission electron microscope equipped with Oxford Inca. X-ray diffraction patterns were performed on a Philips X'Pert Pro Super X-ray diffractometer. Elemental composition of the C-G monolith (10–2.7) was quantitatively determined using an elemental analyser (Vario EL cube, Elementar). Densities were calculated via the weight of C–G monoliths divided by their volumes. BET surface area of C-G monolith (10–2.7) was obtained from the ASAP 2020 Analyser. The electrical conductivity of C–G monolith (10–2.7) was measured using a two-probe method with Keithley 4,200 SCS at room temperature in air. The rebounding process of a metal ball by C–G monolith (15–2.7) was captured by a high-speed video camera (FASTCAM SA5, Photron Limited). The mean recovery speed of C–G monolith was calculated via the displacement of the metal ball divided by the time for the motion between the minimum height of ball and the ball leaves contact with the monolith. *In situ* SEM was performed on MM3A-EM Micromanipulator (Kleindiek Nanotechnik) in Institute of Intelligent Machines, Chinese Academy of Sciences.

### Mechanical testing

Compressive tests were performed using an Instron 5565A equipped with two flat-surface compression stages and 10 N load cells. Cuboidal samples were loaded between the two compression stages with the top stage applying uniaxial compression and release on the samples along the vertical direction. For lamellar structure, the loaded direction was perpendicular to the lamellas. For cellular structure, the loaded direction was perpendicular to the channels. All hysteresis curves were obtained at the strain ramp rate of 0.5 mm s^−1^ with 0% prestrain of the tested samples. For fatigue measurements at 20% strain for 1 × 10^6^ cycles, the interval compression cycles were conducted by using a modal vibrator (ESD-0058, Suzhou Dongling Vibration Test Instrument co., LTD) at 20 Hz. For 2.5 × 10^5^ cycles at 50% strain, the interval compression cycles were conducted by using the modal vibrator at 5 Hz. For 1 × 10^4^ cycles at 80% strain, the interval compression cycles were performed by using Instron 5565A at 0.5 Hz. To evaluate the elastic strength of lamellar C–G monoliths with different microstructures at 50% strain, at least six samples were tested and the average value reported. The vibration of the modal vibrator was induced by an arbitrary waveform generator (DG4000, RIGOL Technologies, Inc.).

### Mechanical simulation

The large geometric deformation, elastic behavior and hysteresis loop of the C–G monolith were investigated by simulating cylindrical thin-shell model using the finite element method. In the simulations, the simply supported boundaries were applied at the straight edges of cylindrical shells to constrain the lateral displacement. For investigating the out-of-plan deformation, the displacement loadings are applied on the arch-shell at different locations in two steps. With regard to elastic strength, the displacement loading is applied on the rigid plane to control the compression and release processes.

A cylindrical shell with simply supported boundary compressed by a rigid plane was used as a simplified mechanical model (Fig. 2c) to investigate the relationship between microstructural and elastic performance of the C-G monolith. We use the thickness *δ* and radius *R*, central angle *θ*, span *x* and height *y* to characterize the geometry shape of the cylindrical shell. Then, we define the depth-span ratio (1):


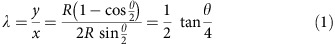


where the depth-span ratio (*λ*) is a function of only central angle. In consideration of the actual parameters of arch microstructures in C–G monoliths, here, we use thickness *δ*=200 nm, radius *R*=20 μm and central angle *θ*=120° as the basic parameters in the simulations. Rigid plane was used to compress and release the cylindrical shell and then calculate the reaction force *F*_KP_ and displacement *U*_KP_ of the KP (key rigid point of rigid plane). The axial length of cylindrical shell is 1 per-unit-length. Therefore, we can get the stress (pressure, *F*_KP_/(1·*x*)) and strain (*U*_KP_/*y*) in the compress-release process. The maximum compression displacement in all of the simulations is half of height *y*. We simulated several compress-release cycles with different geometry parameters with single variable, radius *R* and central angle *θ* and thickness *δ*, respectively. Stresses as a function of strain at different compression cases were then obtained.

For studying the influence of friction action of the laminas in the compress-release process to the hysteresis loop, we use the two opposite cylindrical shells with offset distance Δ*x* (Fig. 2f). Here, in consideration of the abundant wrinkles and little bubbles in the lamellas, we assume the thickness *δ* of cylindrical shell in friction mode as 1 μm, the radius *R* as 20 μm, the central angle *θ* as 120° and the coefficient of friction as 0.3, and we only consider the variation of offset distance Δ*x*. We simulated several different offset distances to investigate its relationship with hysteresis loop. To illustrate the energy loss in the compress-release cycle, we extract the elastic strain energy density profiles of cylindrical shell in one cycle at 20% strain.

### Data availability

The data that support the findings of this study are available on request from the corresponding authors (S.-H.Y. or H.-A.W.).

## Additional information

**How to cite this article:** Gao, H.-L. *et al.* Super-elastic and fatigue resistant carbon material with lamellar multi-arch microstructure. *Nat. Commun.* **7,** 12920 doi: 10.1038/ncomms12920 (2016).

## Supplementary Material

Supplementary Information Supplementary Figures 1-21, Supplementary Table 1 and Supplementary References

Supplementary Movie 1 Dynamic schematic representation of the bidirectional freezing process

Supplementary Movie 2 The carbon elastomer undergoing cyclic compression at 90% strain (captured by a digital camera)

Supplementary Movie 3 The carbon elastomer rebounding a steel ball (captured by high-speed camera)

Supplementary Movie 4 In situ observation of carbon elastomer under compression using scanning electron microscope

Supplementary Movie 5 In situ observation of single arch-shell under compression using scanning electron microscope
