# Supplementary material for: Super-elastic and fatigue resistant carbon material with lamellar multi-arch microstructure
Source: Nat Commun. 2016 Sep 27;7:12920. doi: 10.1038/ncomms12920 (PMC5052633; doi:10.1038/ncomms12920)
Supplement: Supplementary Information — Supplementary Figures 1-21, Supplementary Table 1 and Supplementary References [file ncomms12920-s1.pdf]

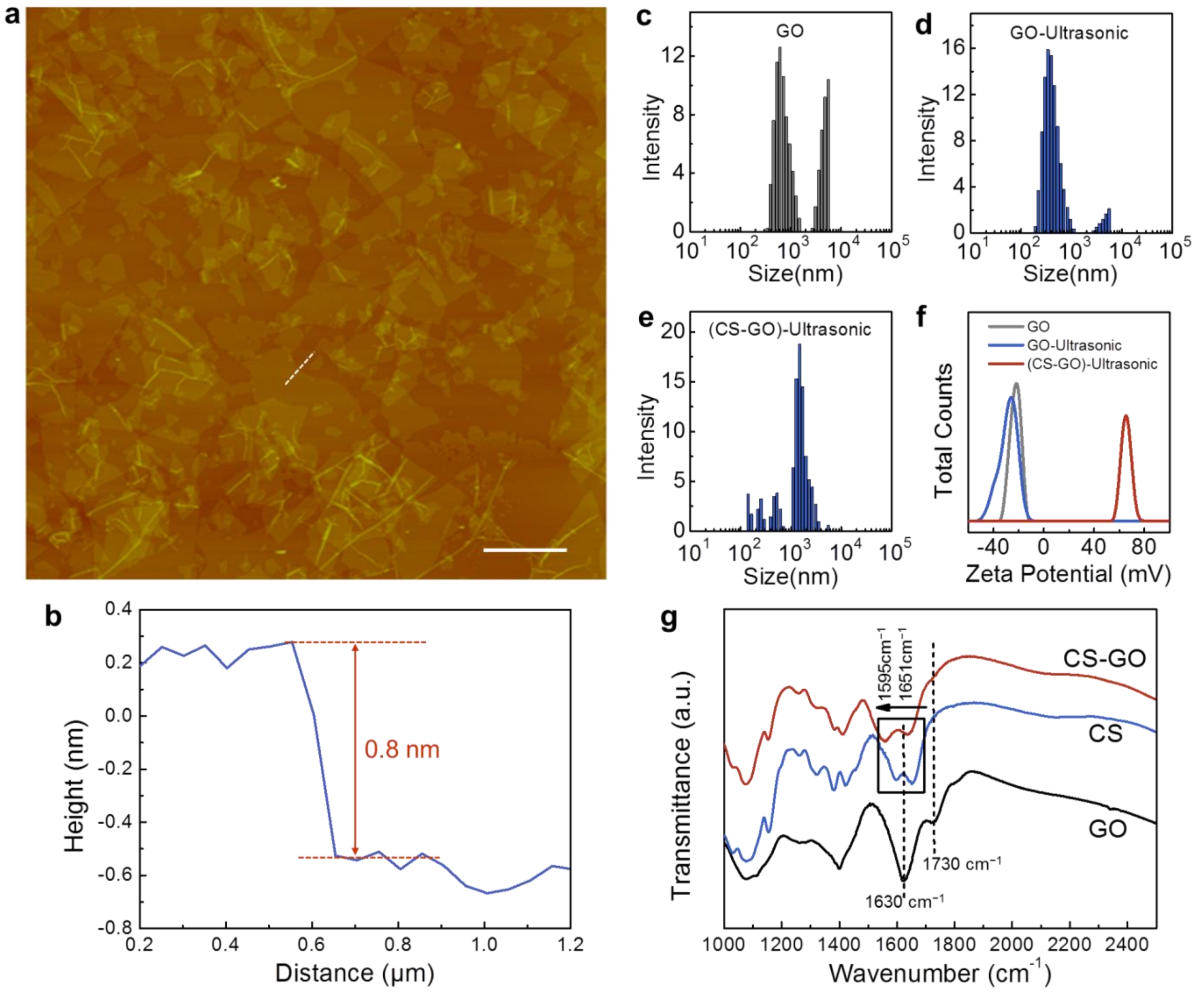

Supplementary Figure 1│Characterizations of the raw materials. **a**, **b**, Atomic force microscopy (AFM) image and its corresponding height profile (the dotted line) show that the graphene oxide (GO) sheets on a silica surface are flat (~0.8 nm thick). Scale bar, 5 μm. **c-e**, Size distribution of GO sheets before and after coating with CS show that GO sheets are dispersed uniformly in CS solution. We notice that, the size of GO sheets reduced after ultrasonic treatment. **f**, Zeta potential profiles show that positively charged CS molecular chains are fully coated on the surface of negatively charged GO sheets. **g**, FTIR spectra show that interaction between GO and CS occurs. The peak at 1730 and 1630 cm^{-1} in the spectrum of GO are characteristics of the C=O stretch of the carboxylic group on the GO and deformations of the O-H bond, respectively. Two characteristic absorbance bands at 1651 and 1596 cm^{-1} in the spectrum of CS correspond to the C=O stretching vibration of -NHCO- and the N-H bending of $-NH_2$, respectively. Notably, in the spectrum of CS-GO, C=O stretch of the carboxylic group at 1730 cm^{-1} disappears and both the peaks of C=O stretching vibration of -NHCO- and the N-H bending of $-NH_2$ shift to lower wavenumbers. These results reveal that hydrogen bonding between CS and the oxygenated groups in GO as well as electrostatic interaction between positive charge of CS chains and the negative charge on the surface of GO should happen[1].

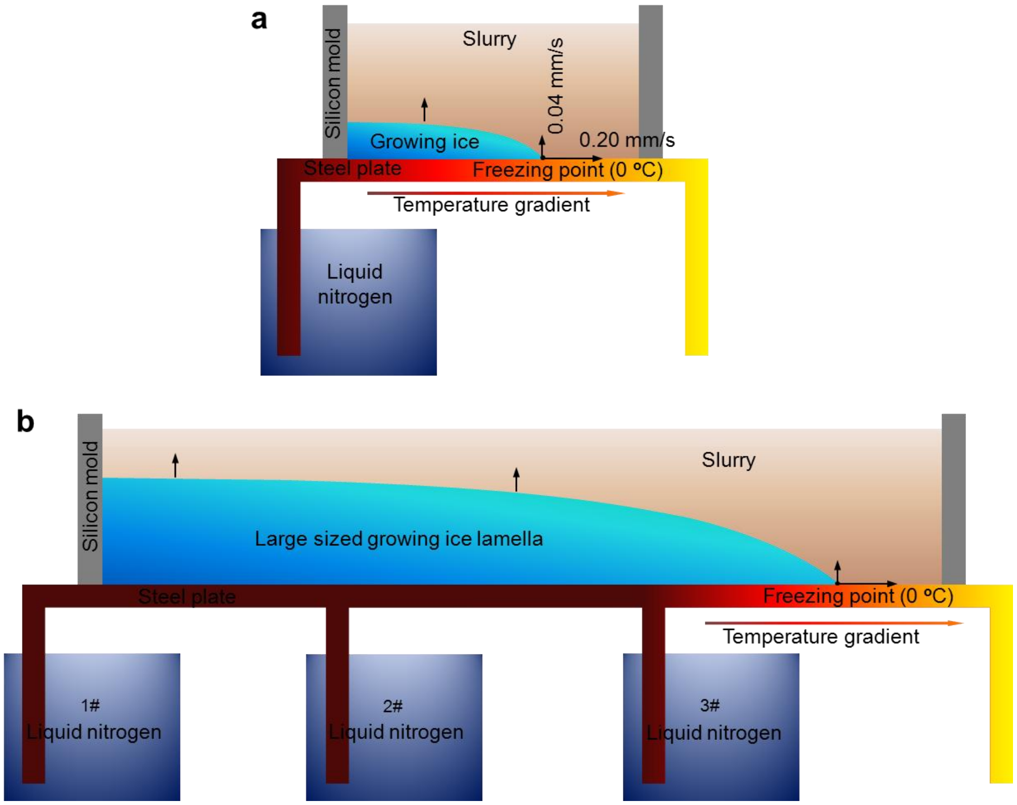

Supplementary Figure 2 | Schematic illustrations of bidirectional freezing. a, Schematic illustration shows the details in the developed bidirectional freezing process. **b**, Schematic illustration reveals the feasibility of this bidirectional freezing for fabricating large-sized monolithic material. In this bidirectional freeze casting method, we control ice crystals grow along both horizontal direction and vertical direction by cooling the freezing plate from one end to the other end and make a temperature gradient on the plate surface, driving the freezing points move from one end to another end on the freezing surface. Thus, the initial formed ice nucleus would grow with the moved freezing points and form long-range paralleled ice columns. These paralleled ice columns simultaneously grow along vertical direction to form long-range paralleled ice lamellas. It is worth noting that this freezing method has simpler process, lower equipment requirement and is more beneficial for fabricating large-sized long-rang parallel lamellar structure.

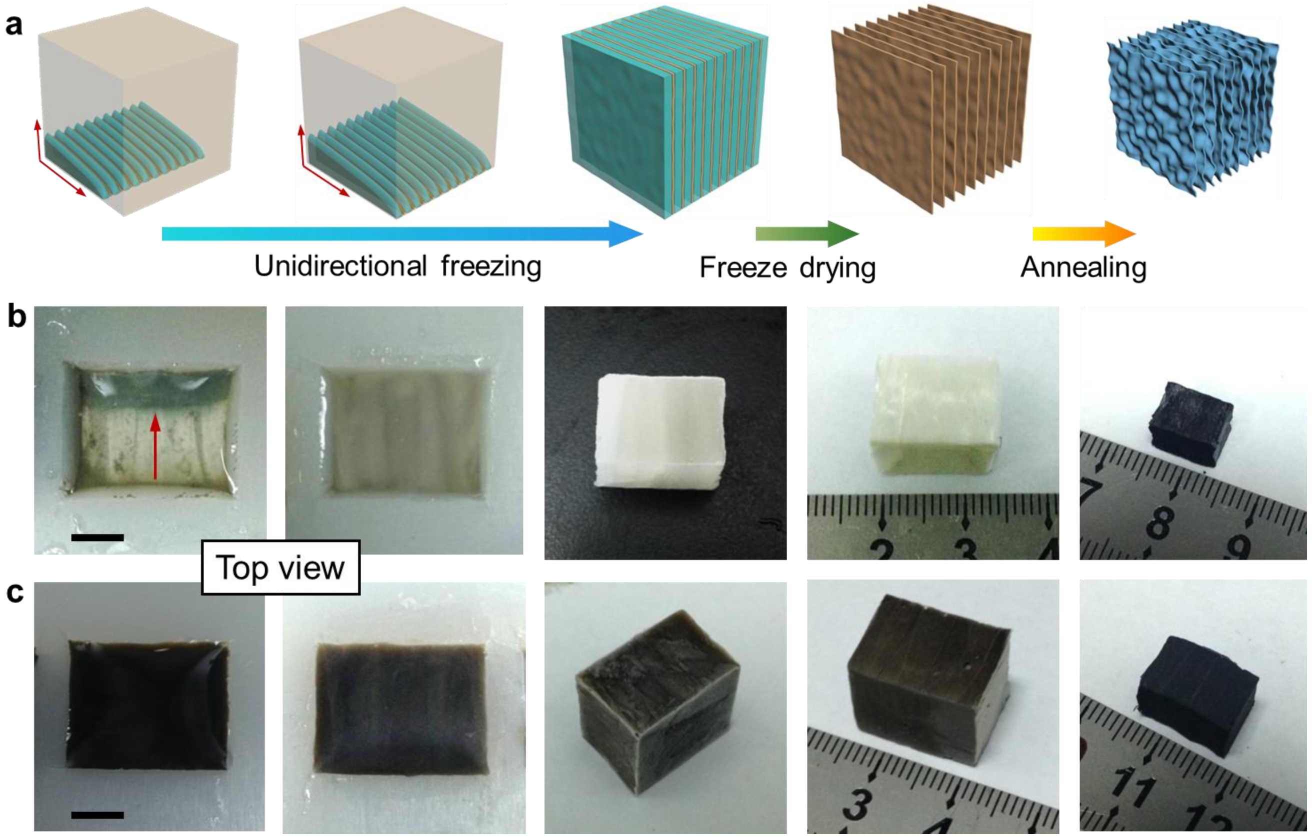

Supplementary Figure 3│Materials preparation process. **a**, Schematic illustrations show the process of bidirectional freezing combined with annealing treatment. **b**, Digital photographs show the fabrication process of lamellar carbon (C) monolith from pure chitosan (CS). Scale bar, 5 mm. **c**, Digital photographs show the fabrication process of lamellar (carbon-graphene) C-G monolith from CS-GO composite. In this process, the CS-GO was separated and put together between the adjacent ice lamellas. After freeze drying, bulk CS-GO scaffolds composed of centimeter-range paralleled lamellas can be obtained. Scale bar, 5 mm.

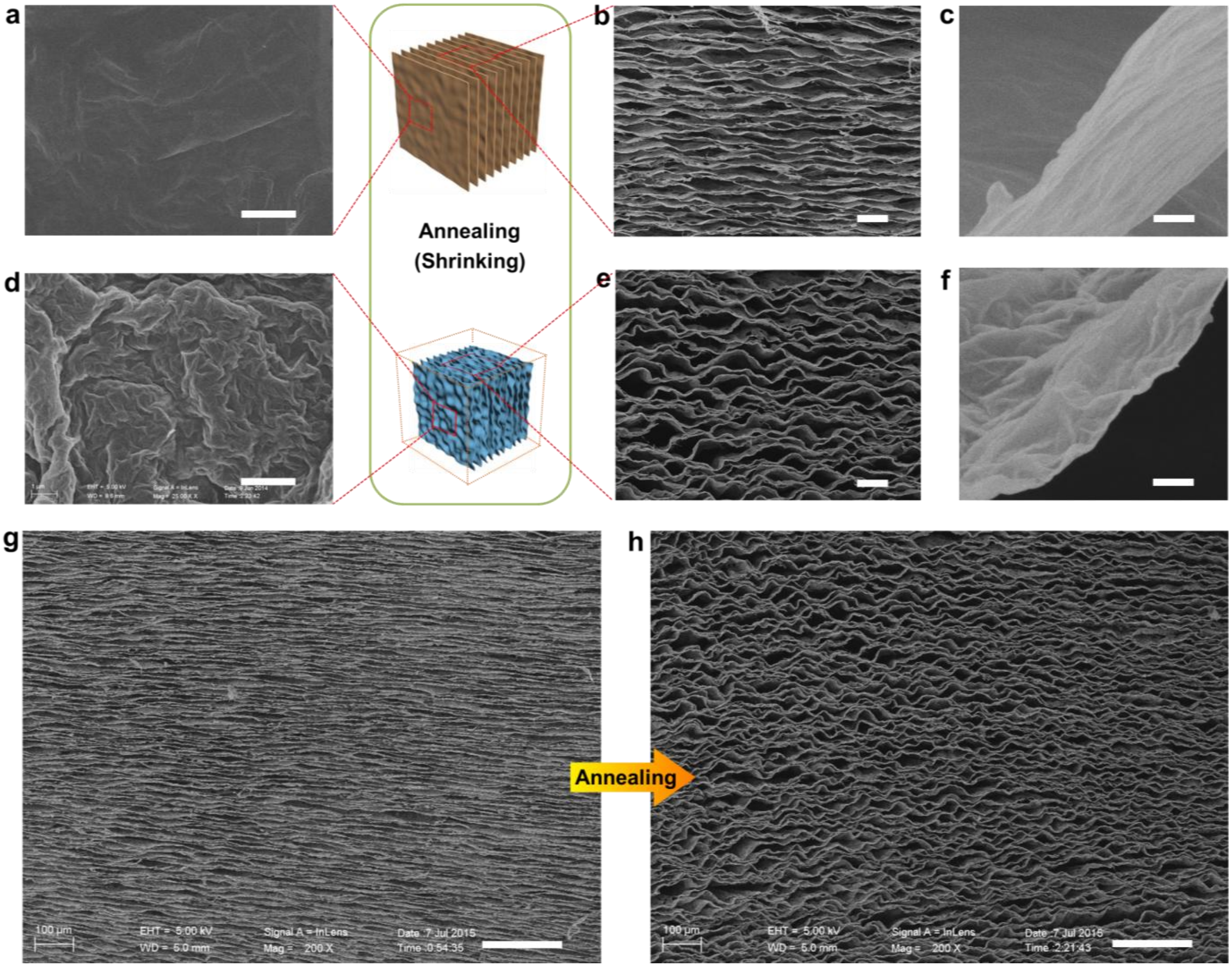

Supplementary Figure 4 | Microstructural change of the monolithafter annealing treatment. a-**c**, Scanning electron microscope (SEM) images show the relatively flat lamellas with nanometer thickness in CS-GO scaffold before annealing treatment. Scale bars, 2 μm (**a**), 50 μm (**b**), 500 nm (**c**). **d**-**f**, SEM images show the wrinkled and waved morphology of the lamellas in C-G monolith after annealing treatment. Scale bars, 2 μm (**d**), 50 μm (**e**), 500 nm (**f**). **g**,**h**, SEM images show long-range paralleled lamellar structure of CS-GO scaffold with relatively flat lamellas (**g**) and C-G monolith with waved lamellas (**h**). Scale bars, 200 μm for both.

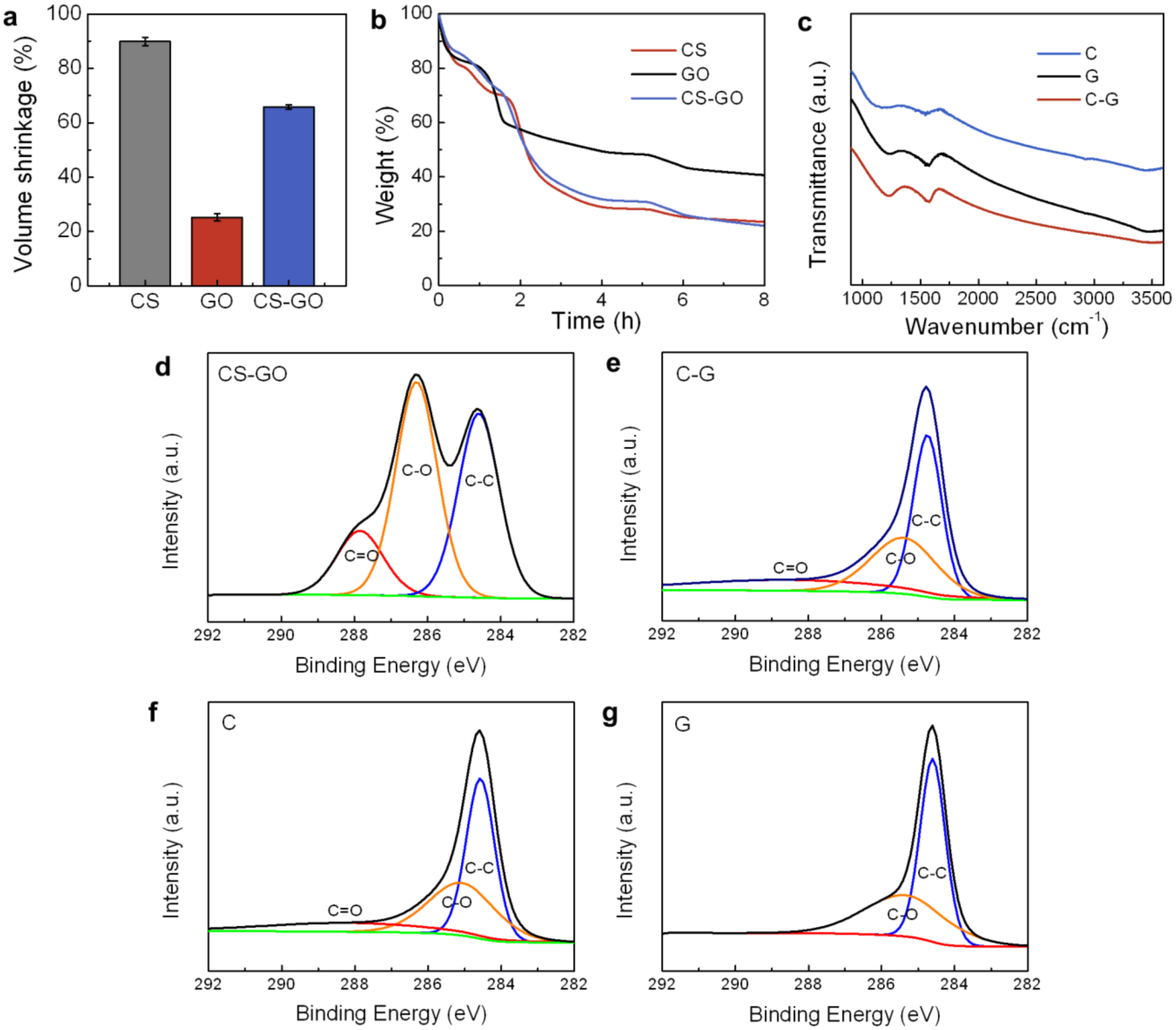

Supplementary Figure 5│Characterizations of the obtained monolithic materials. a, Volume shrinkage of CS, GO and CS-GO monoliths after annealing treatment with the same condition (800 ^{o}C for 2 hours in N_2). All the error bars represent the s.d. of at least six replicate measurements. **b**, Thermal gravity analysis (TGA) reveals the weight loss in the annealing treatment of CS, GO and CS-GO monoliths. Both the volume shrinkage and weight loss of CS monoliths are much larger than GO monoliths. **c**, Fourier transform infrared spectroscopy (FTIR) spectrums show that CS, GO and CS-GO monoliths all loss most of their functional groups in the high temperature annealing process. **d**-**g**, X-ray photoelectron spectroscopy (XPS) C1s spectra of CS-GO, C, C-G and G monolith show that the annealing treatment removes large amount of C-O and C=O groups in these materials.

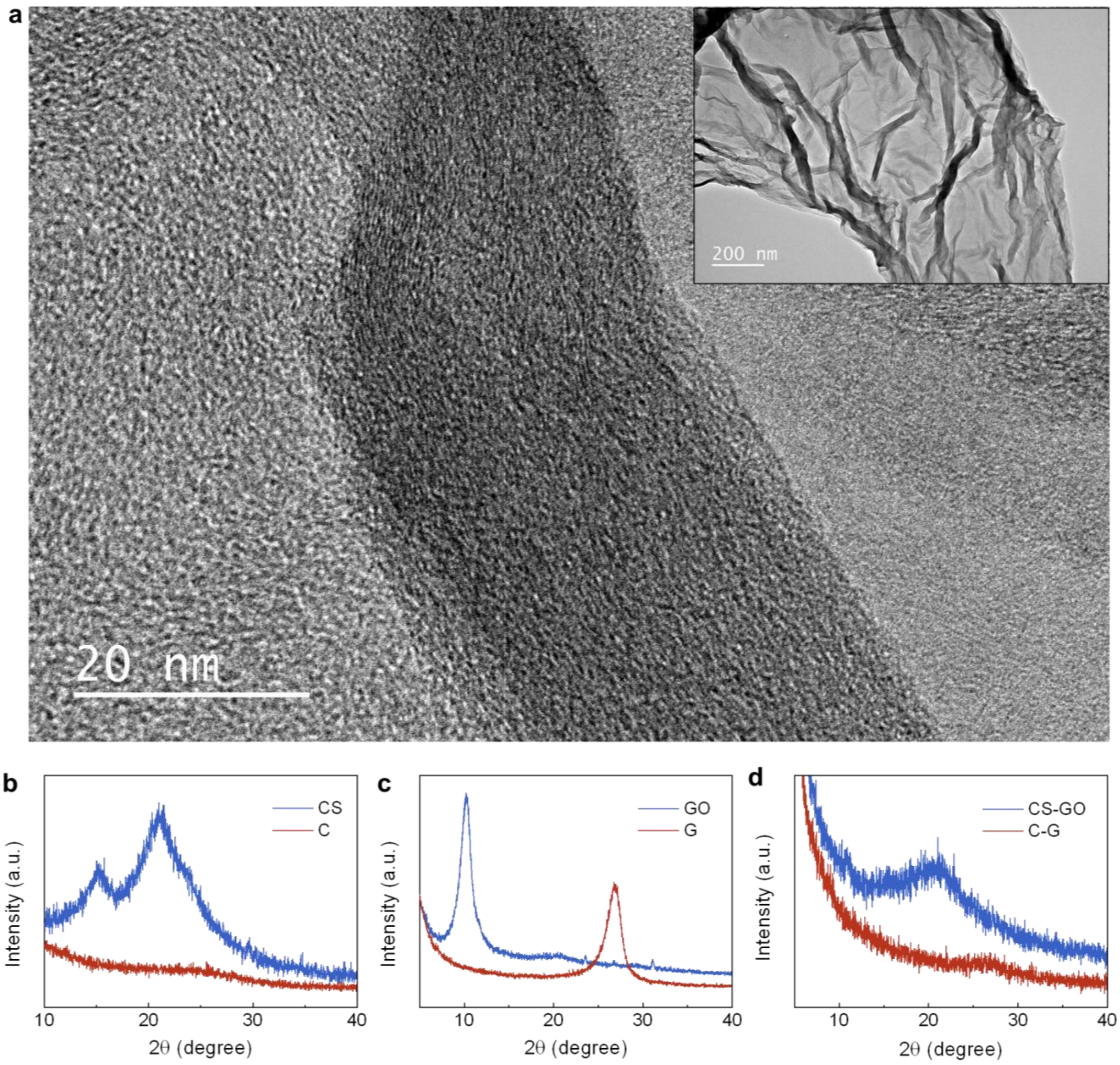

Supplementary Figure 6│Characterizations of the obtained monolithic materials. a, Typical transmission electron microscopy (TEM) image shows the wrinkled lamella (**insert**) and high resolution transmission electron microscopy (HRTEM) image shows that the lamella is composed of amorphous carbon and graphene composite. No apparent boundary between them can be seen, which certified that amorphous carbon and graphene are tightly integrated together. **b-d**, X-ray diffraction (XRD) patterns of CS, GO and CS-GO monoliths before and after annealing treatment reveal that the XRD pattern of the CS-GO monolith shows only the CS diffraction peaks from CS and the diffraction peak of GO disappears. The result demonstrates that GO are mainly exfoliated as individual sheets in the polymer matrix and loss the regular and periodic structure of graphene. The broad weak diffraction peaks at ca. 25° in (**b**) and (**d**) indicate that CS is converted into amorphous carbon after annealing treatment.

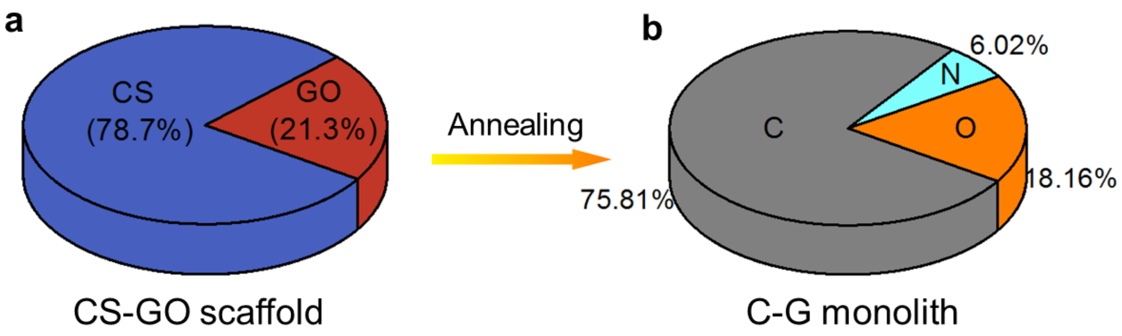

Supplementary Figure 7│Constituents analysis of the monoliths. a, The calculated weight percentage of the two constituents in CS-GO (10-2.7) monolith before annealing treatment. **b**, Organic elemental analysis displays that the ultimate C-G monolith were mainly composed of carbon.

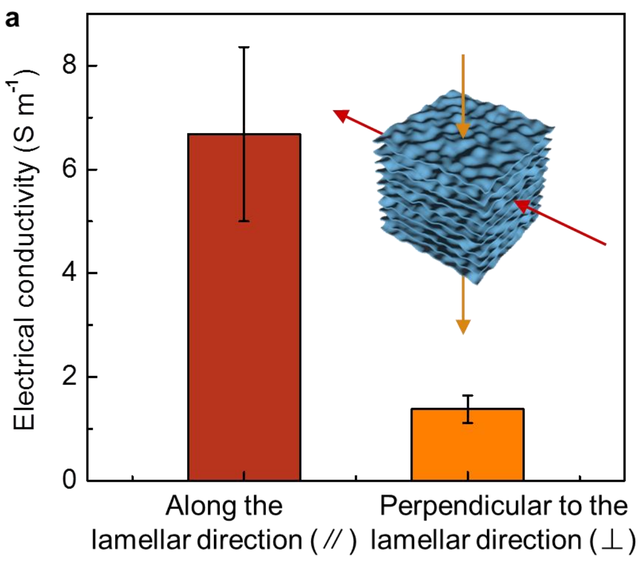

Supplementary Figure 8 | Electrical conductivity of the carbon monoliths. a, The electrical conductivity of C-G monoliths along the lamella direction and perpendicular to the lamella direction are totally different. The error bars represent the standard deviations of three replicate measurements. **b**, Comparison of the electrical conductivity of C-G monoliths as a function of density to several previously reported low-density carbon materials[2-14]. Numbers in the charts represent relevant references.

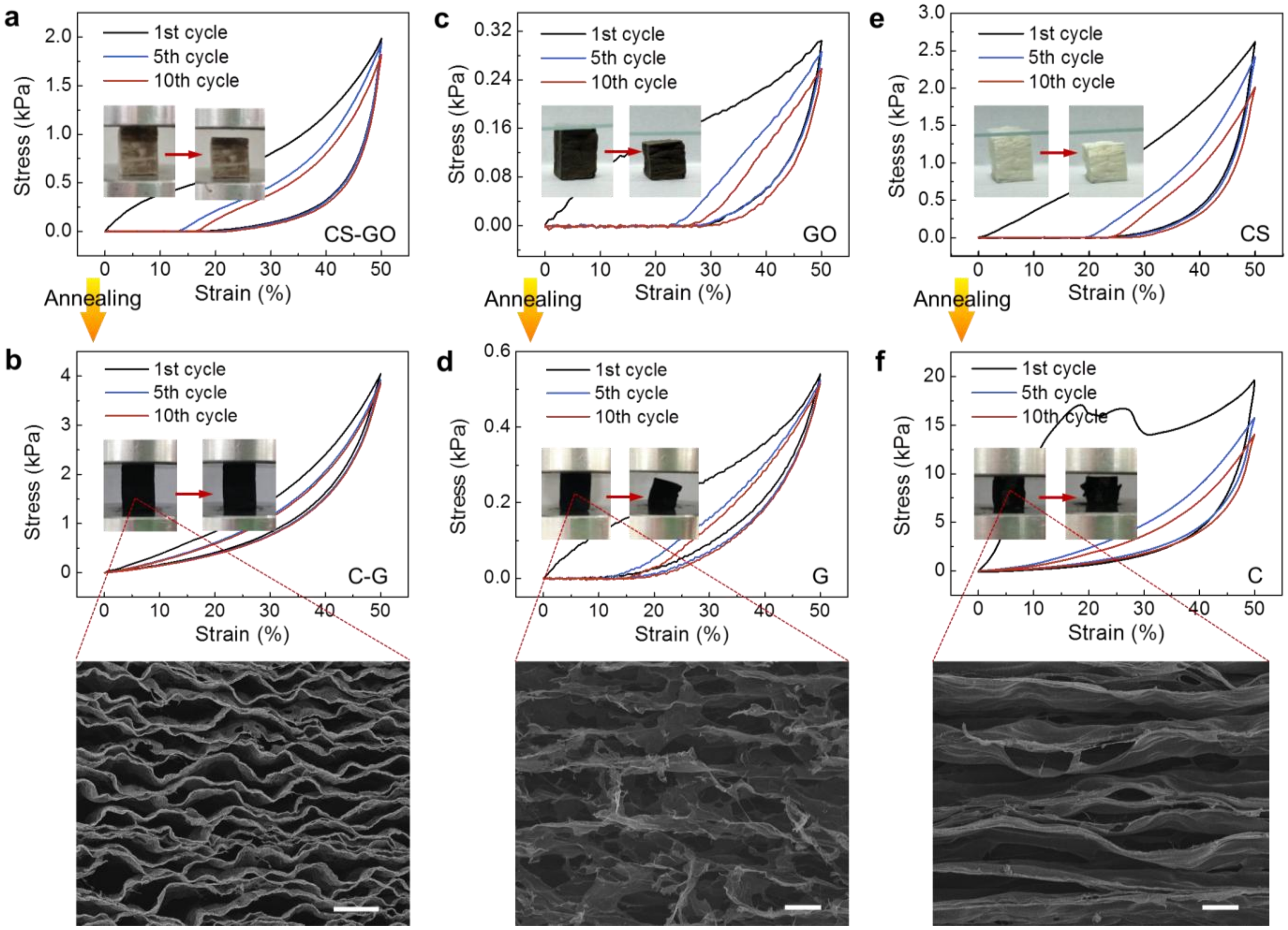

Supplementary Figure 9│Mechanical behavior comparison. **a**, Stress-strain curve of lamellar CS-GO monolith shows that it exhibits weak elasticity, large hysteresis loop and obvious plastic deformation. The insert digital photos indicate that it can't recover to its original height after the first compression cycle. **b**, Stress-strain curve of lamellar C-G monolith shows that it is highly elastic and displays a narrow hysteresis loop after the first compression cycle. The insert digital photos indicate that no permanent deformation occurs. **c**,**d**, Stress-strain curves and insert digital photos show that lamellar GO monolith display inferior elasticity and large plastic deformation before and after annealing treatment **e**,**f**, Stress-strain curves and insert digital photos show that lamellar CS monolith is converted into a very brittle lamellar AC monolith after carbonization. Scale bars, 50 μm (**b**), 10 μm (**d**), 20 μm (**f**).

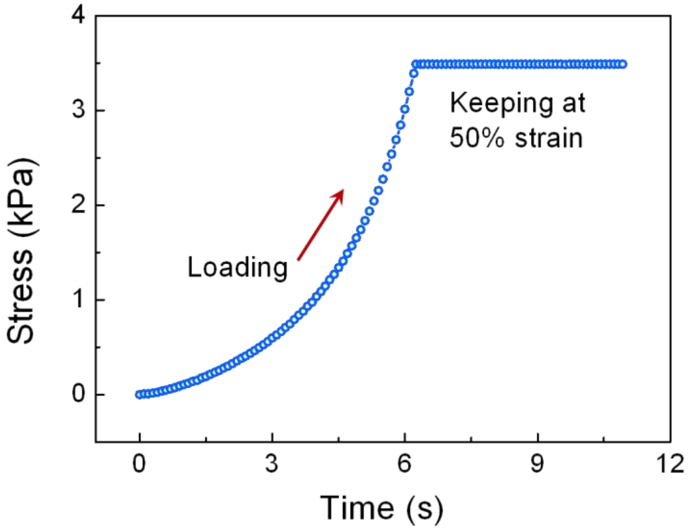

Supplementary Figure 10 | Stress-time curve in static compression test. Stress as a function of time in a static compression test shows that C-G monolith was compressed to 50% strain and kept for a few seconds. The elastic strength maintained at a constant value without diminishing when the compression strain was keeping steady.

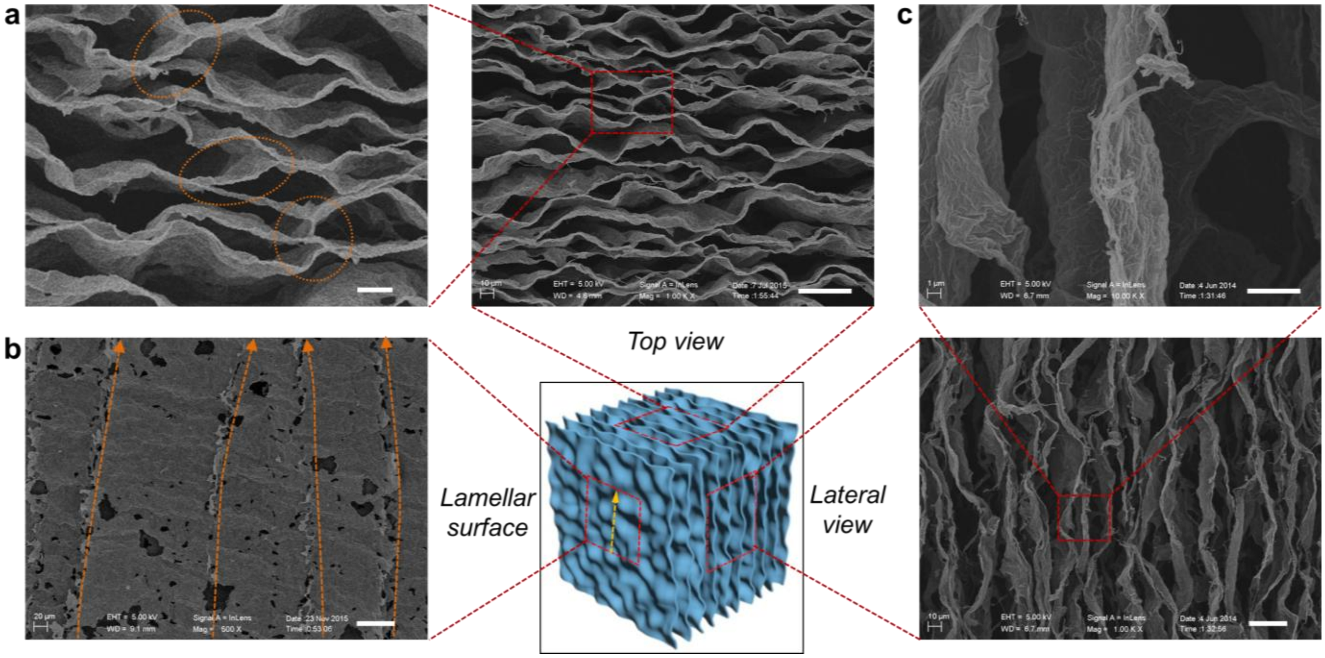

Supplementary Figure 11│Microstructural details of the monolith. a, SEM images from the top view show that the lamellas have waved multi-arch morphology and there are some randomly distributed bridge ligaments (marked in yellow circles) linking two adjacent lamellas. Scale bars, 10 µm, 40 µm, from left to right. **b**, SEM image of the lamella surface shows that the ligaments are parallel to the vertical direction, and the distance between two adjacent ligaments is rang from several ten to several hundred of micrometers. The ligaments should arise from the interrupt of the growing ice columns in the initial freezing process. The interrupted ice columns then grow into interrupted ice lamellas with CS-GO trapped between them to form the bridge ligaments. Scale bar, 50 µm. **c**, SEM images show the lamellas from the lateral view. Scale bars, 4 µm, 50 µm, from up to down.

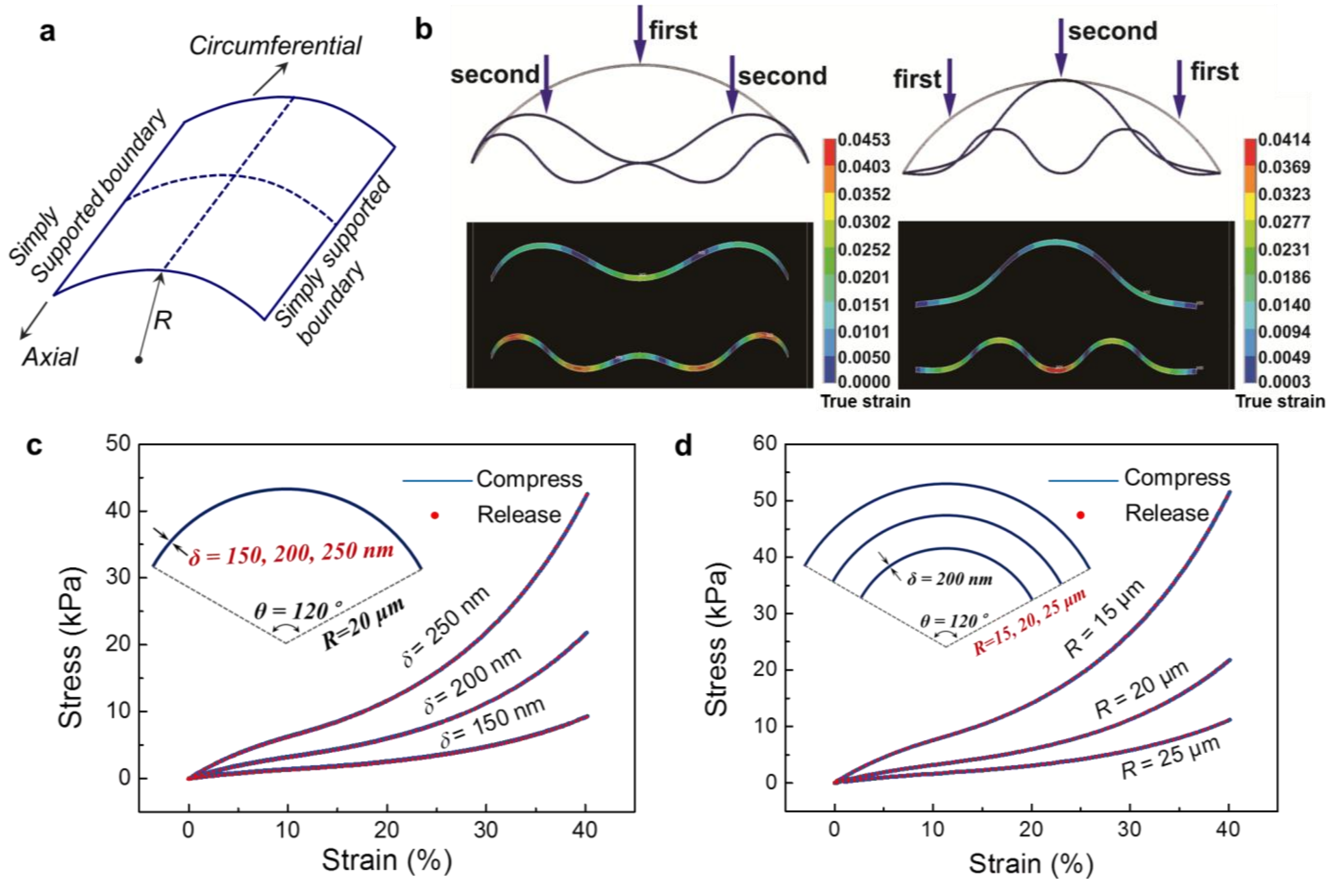

Supplementary Figure 12│Mechanical simulations of cylindrical shell models. **a**, Schematic diagram shows cylindrical shell model with simply supported boundary. **b**, Schematic illustrations show the large geometry deformation of the cylindrical shell modes. In order to explain the large elastic compression of C-G monish in the experiment, we performed finite element method to simulate the thin-arch-shells with large geometric deformation. The two different structural deformations induced by different loading sequences show they are under large out-of-plan deformation without structural yielding. The blue arrows on the shell represent the loading locations and sequences. The waved shapes of the two models indicated that both the two arch-shells have a large deformation. Whereas the maximum true strain (von Mises total mechanical strain) values for the two cases were as small as 0.045 and 0.041, respectively. **c**, **d**, Stress-strain curves in compress-release cycle of the single cylindrical shell models with different parameters and schematic diagrams show the corresponding parameters.

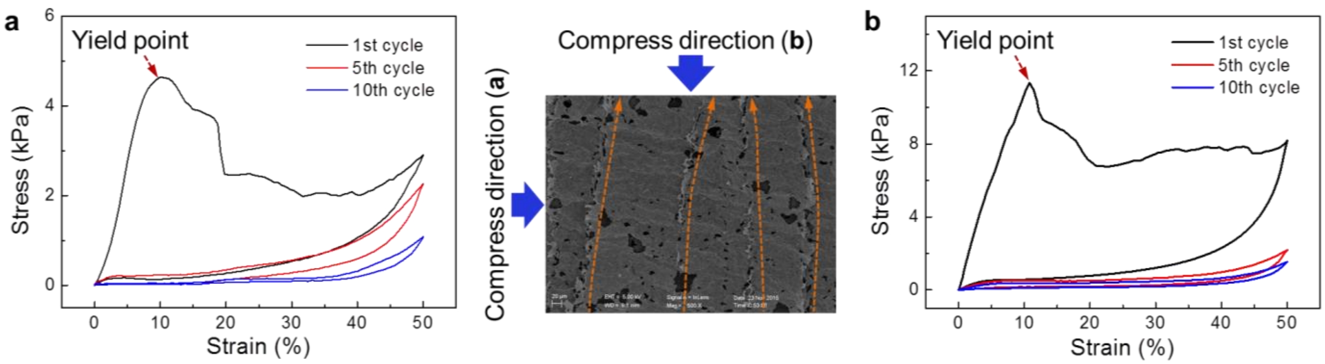

Supplementary Figure 13 | Compression tests of the carbon monoliths at different directions. a,**b**, Stress-strain curves of C-G monoliths under compression from two lateral directions. C-G monoliths in both the lateral directions reveal typical brittle behavior and are easily to be damaged when undergo compression in these two directions. This result is distinct to that observed for the direction perpendicular to the lamellas.

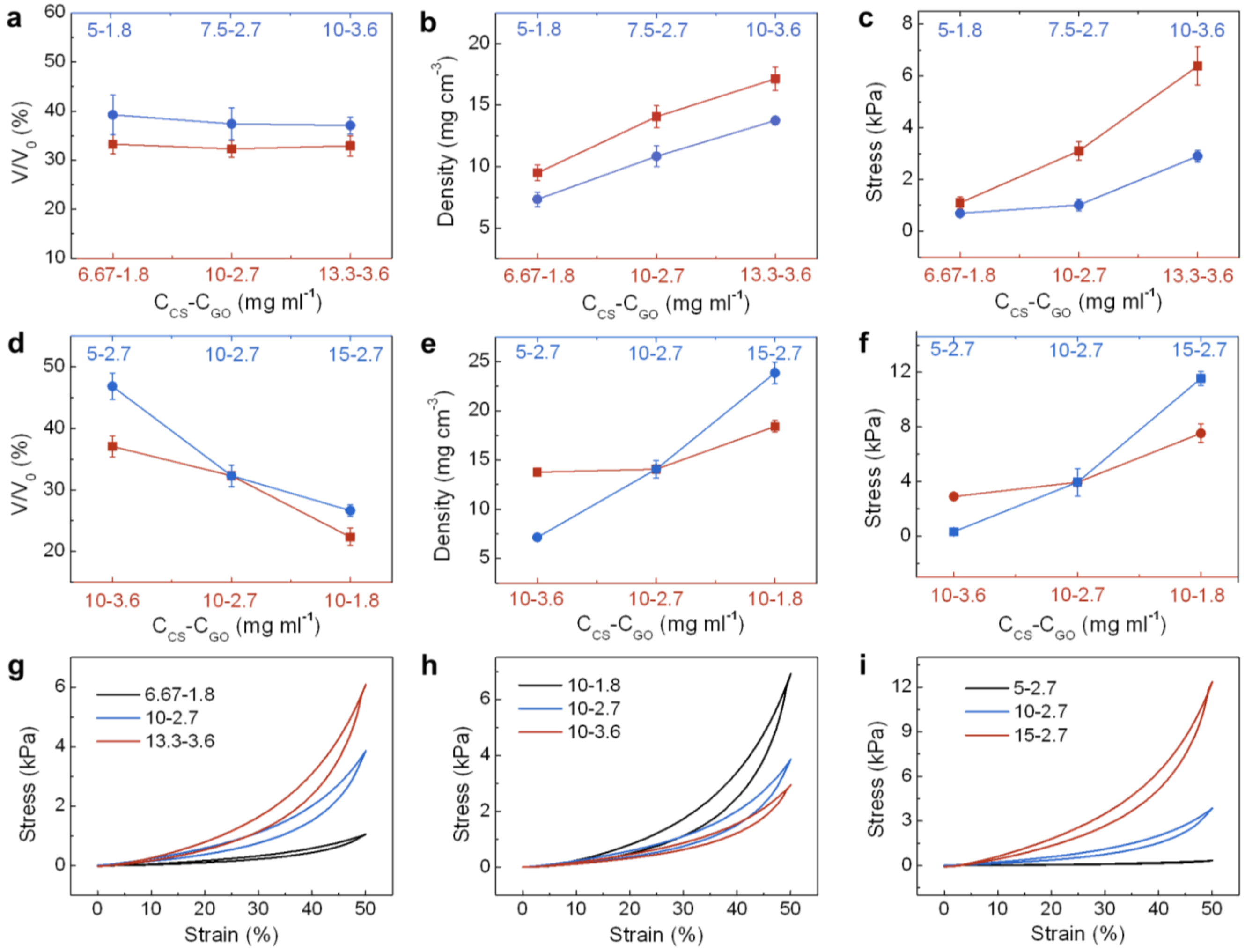

Supplementary Figure 14 │ Characterizations of the obtained monolithic materials. a, The volume shrinkage of CS-GO scaffolds after annealing treatment was nearly equal when the initial mass ratio of CS and GO was constant. **b**, When the ultimate volume of the C-G monoliths was nearly equal, the densities increase with the increasing of the initial CS and GO contents. **c**, When the volume shrinkage was nearly equal, the maximum stress at 50% strain of C-G monoliths increase with increasing of the initial CS and GO contents, which should be attributed to the increase of the lamella thickness of C-G monoliths. **d**, When CS content is constant, volume shrinkage of C-G monoliths increases with the decreasing of GO. When GO content was constant, volume shrinkage of C-G monoliths increases with increasing of CS. **e**, Density of C-G monoliths is determined by both the content and initial mass ratio of CS and GO. **f**, The maximum stress at 50% strain of C-G monoliths are also determined by both the content and initial mass ratio of CS and GO. (**g-i**) Stress-strain curves of C-G monoliths made from CS and GO with different mass ratio. All the error bars represent the s.d. of at least six replicate measurements. C_{CS}-C_{GO} represents the concentration of CS and GO in the initial CS-GO composite suspensions for fabricating the C-G monoliths.

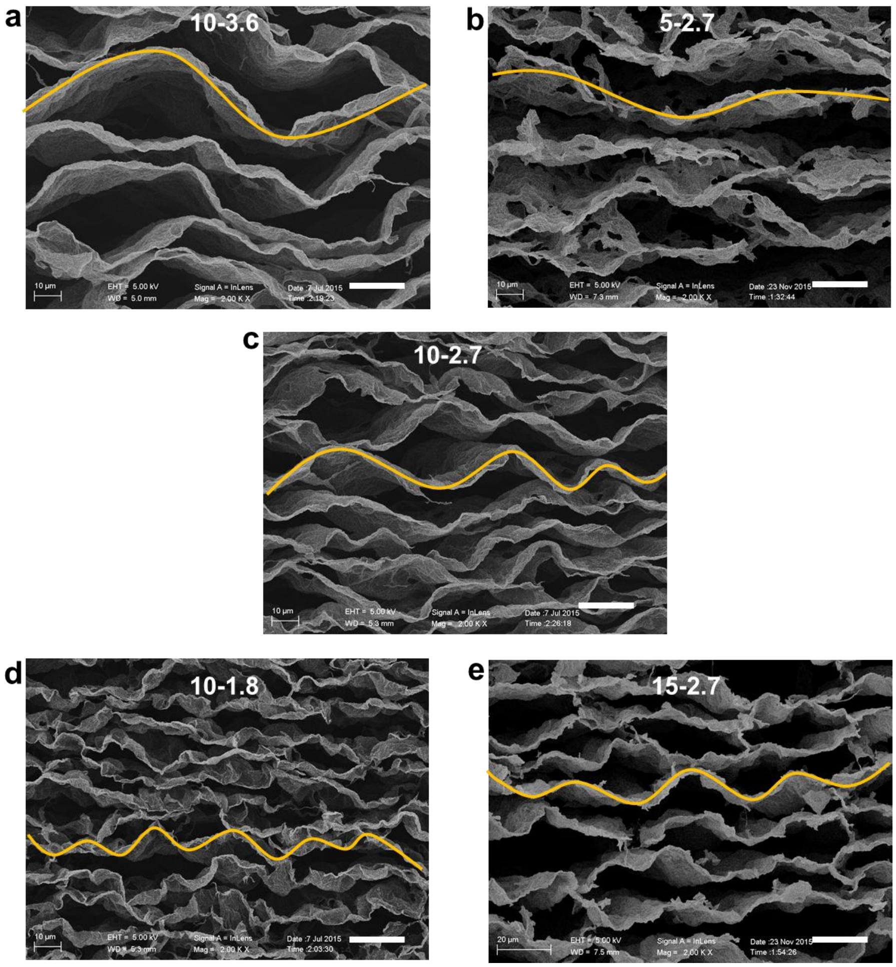

Supplementary Figure 15 | Microstructural comparison. a-e, SEM images show the top view of C-G monoliths made from CS and GO with different mass ratio. The results reveal that the mass ratio of initial CS and GO has big effect on the shrinking of the lamellas. Larger mass ratio (CS-GO) results in lamellas with smaller arch microstructure. Scale bars, 20 μm for all.

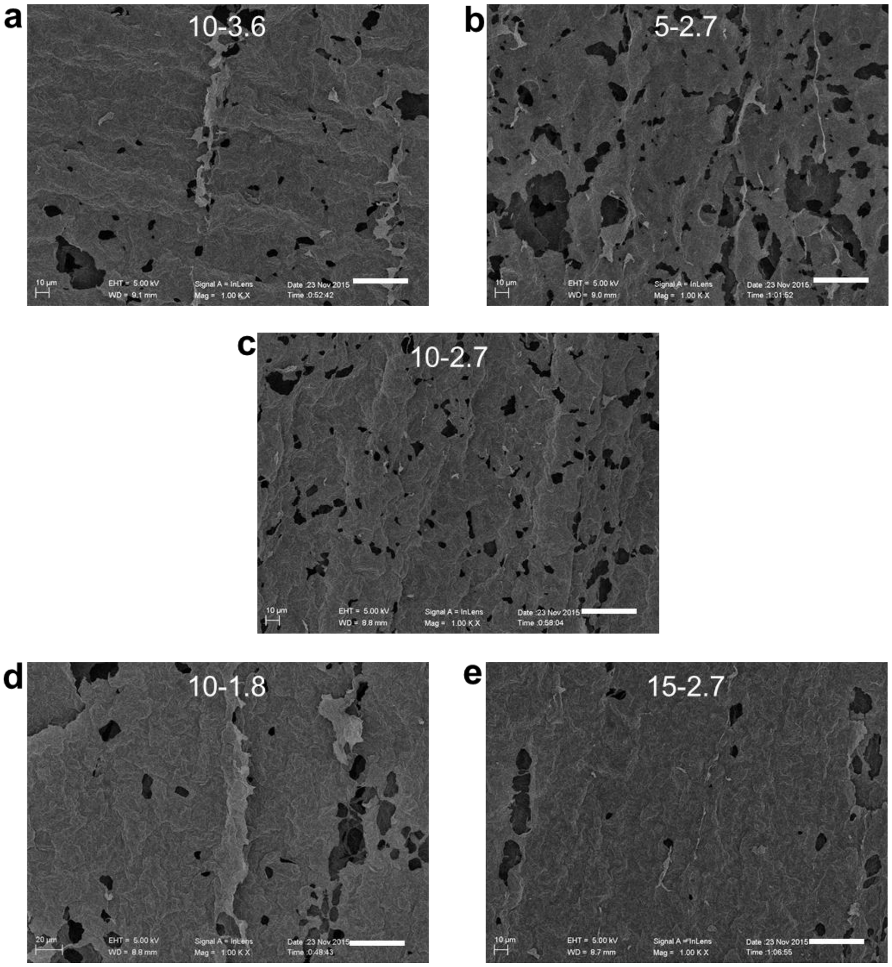

Supplementary Figure 16 | Microstructural comparison. a-e, SEM images show the lamella surface of C-G monolith made from CS and GO with different mass ratio. The results reveal that the integrity (thickness) of the lamellas is mainly determined by the initial CS content. When the GO content is constant, increasing CS would increase the integrity (thickness) of the lamellas, and the number of defects in the lamellas would decrease. While, the initial GO content has little effect on the lamella integrity. Scale bars, 40 μm for all.

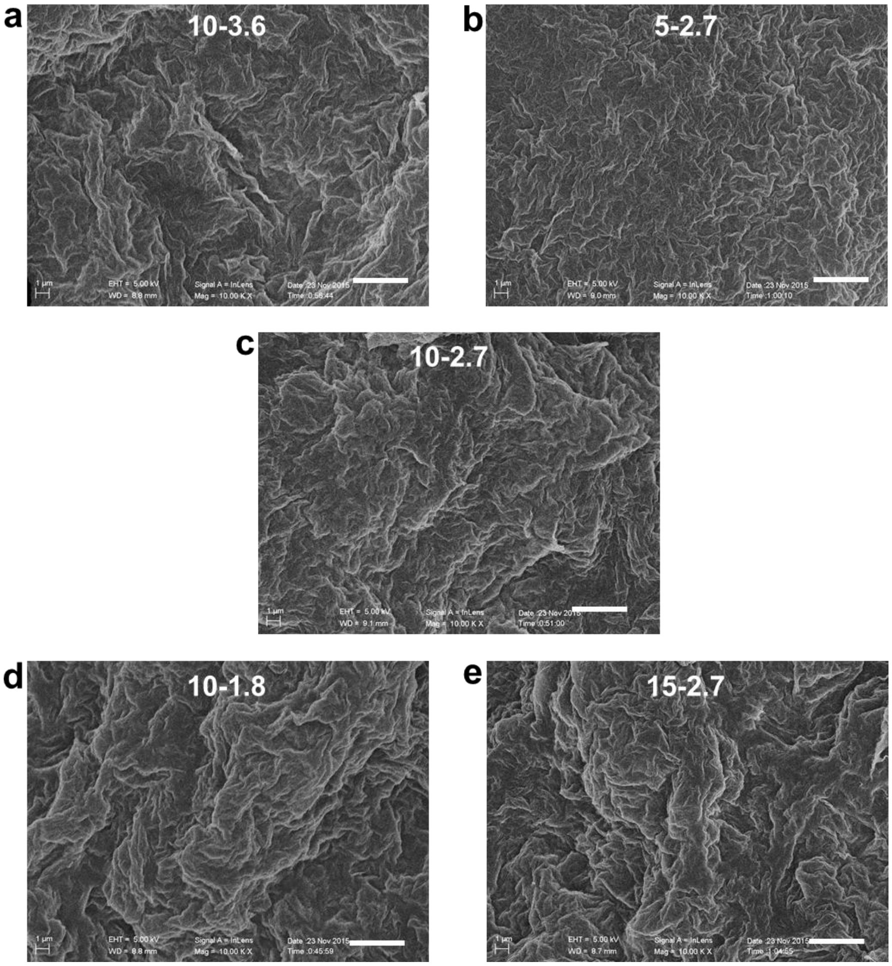

Supplementary Figure 17│Microstructural comparison. a-e, SEM images with higher magnification show the lamella surface of C-G monolith made from CS and GO with different mass ratio. The results reveal that the mass ratio of initial CS and GO has big effect on the shrinking of the lamellas. Larger mass ratio of CS-GO would result in larger level of shrinkage of the lamellas. Scale bars, 4 μm for all.

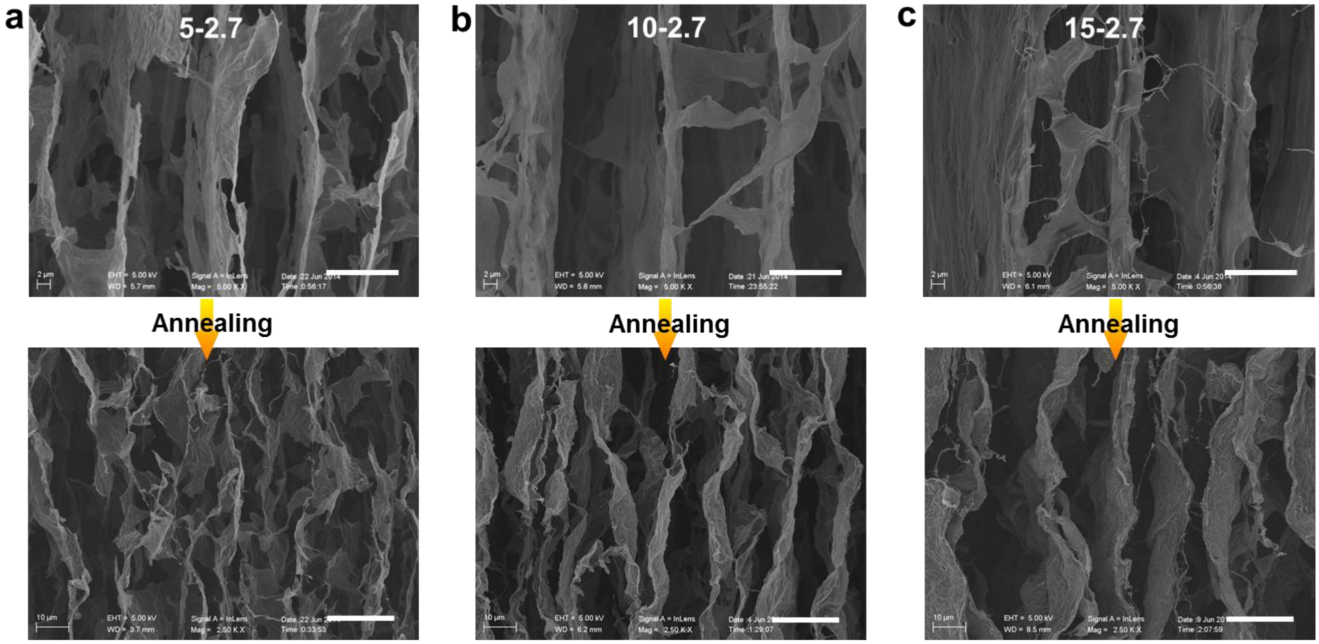

Supplementary Figure 18 | Microstructural comparison. a-c, SEM images show the cross section of CS-GO scaffold with different mass ratio of CS and GO before and after annealing treatment. The results reveal the influence of initial CS content to the thickness of the lamellas. When the GO content is constant, increasing CS would increase the thickness of the lamellas, and the number of defects in the lamellas would decrease. Scale bars, 10 μm for up, 20 μm for down.

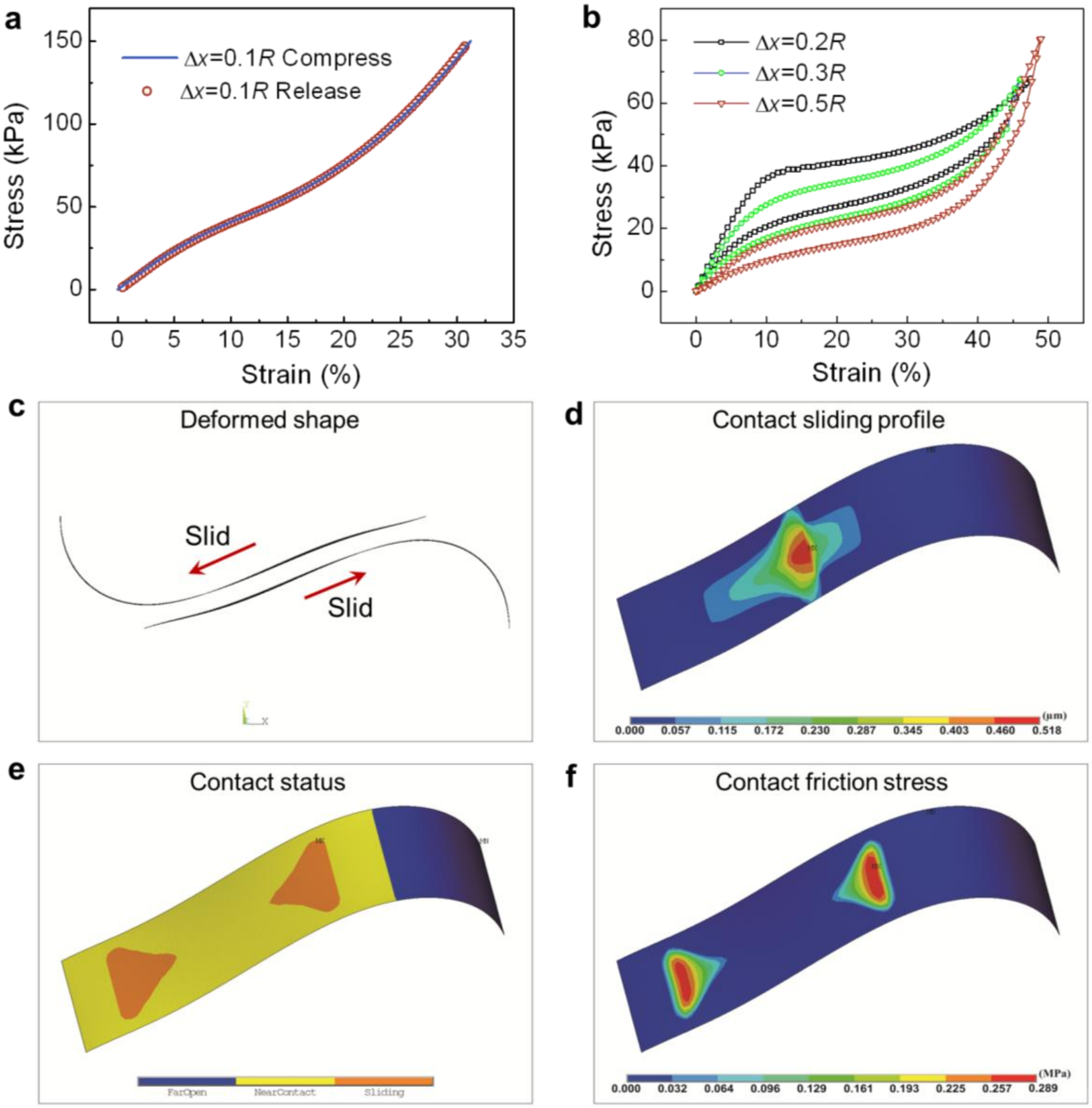

Supplementary Figure 19 | Mechanical simulations of two opposite cylindrical shells. a, **b**, Stress-strain curves of two opposite cylindrical shells with different stagger distance Δ*x* in the compress-release cycle. **c**, Deformed shape of the two opposite cylindrical shells. **d**, Total contact sliding profile of the contact area. **e**, The contact status of contact area at current state. **f**, The contact friction stress of contact area at current state. For (**c**-**f**), the stagger distance Δ*x* is 0.4*R*, the strain is about 50%. The simulated results demonstrated that there was no hysteresis loop in the stress-strain curve with small Δ*x* (Δ*x*=0.1*R*) because no relative sliding happened. For larger Δ*x* (Δ*x*=0.2-0.5*R*), the two cylindrical shells began to slide to each other.

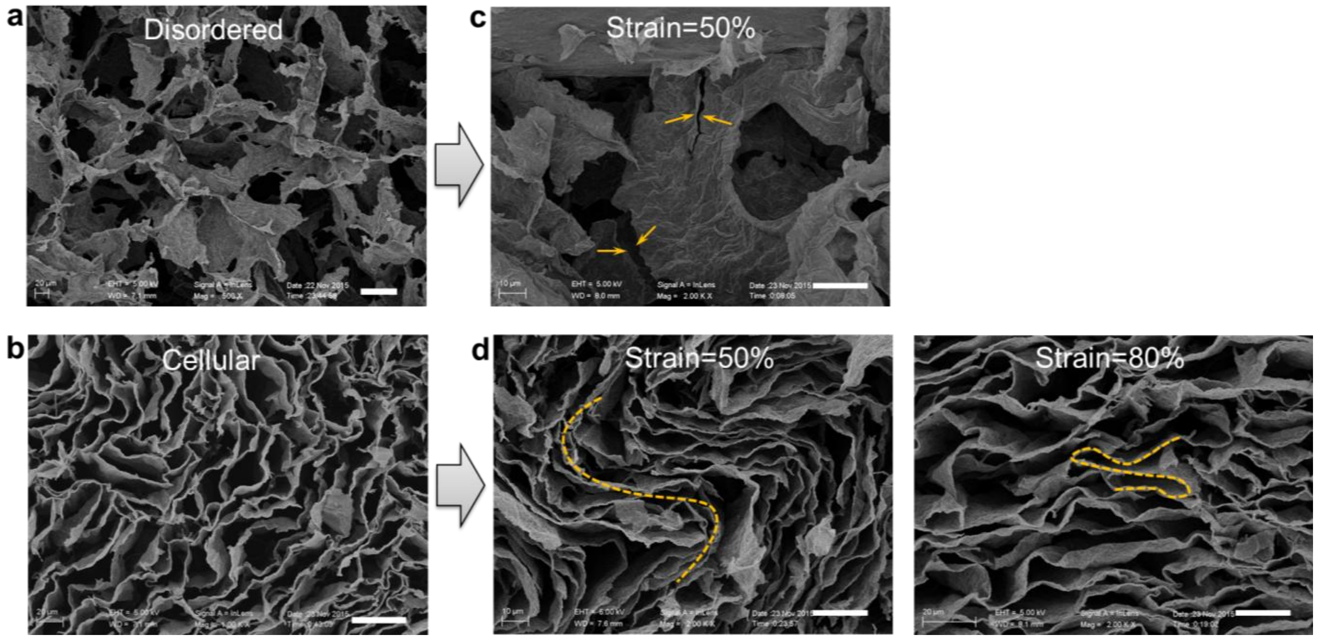

Supplementary Figure 20 | Microstructure of disordered and cellular monoliths. a,b, SEM images show the top view of C-G monoliths with disordered structure and cellular structure. Scale bars, 50 μm (**a**), 40 μm (**b**). **c,** SEM observation shows that a lot of structural fractures happen in the disordered C-G monolith at 50% compression strain. Scale bar, 20 μm. **d,** SEM observation shows that cellular walls mainly undergo bending instead of buckling failure in the cellular C-G monolith at 50% strain. So, its structure is still stable. However, when it suffers from continuing compression with larger strain (80%), its cellular walls begin to fold and undergo buckling failure. Repeatedly occurring of these cases under cyclic compression would inevitably lead to the subsequent strength reduction, plastic deformation and large energy dissipation. Scale bars, 20 μm for both.

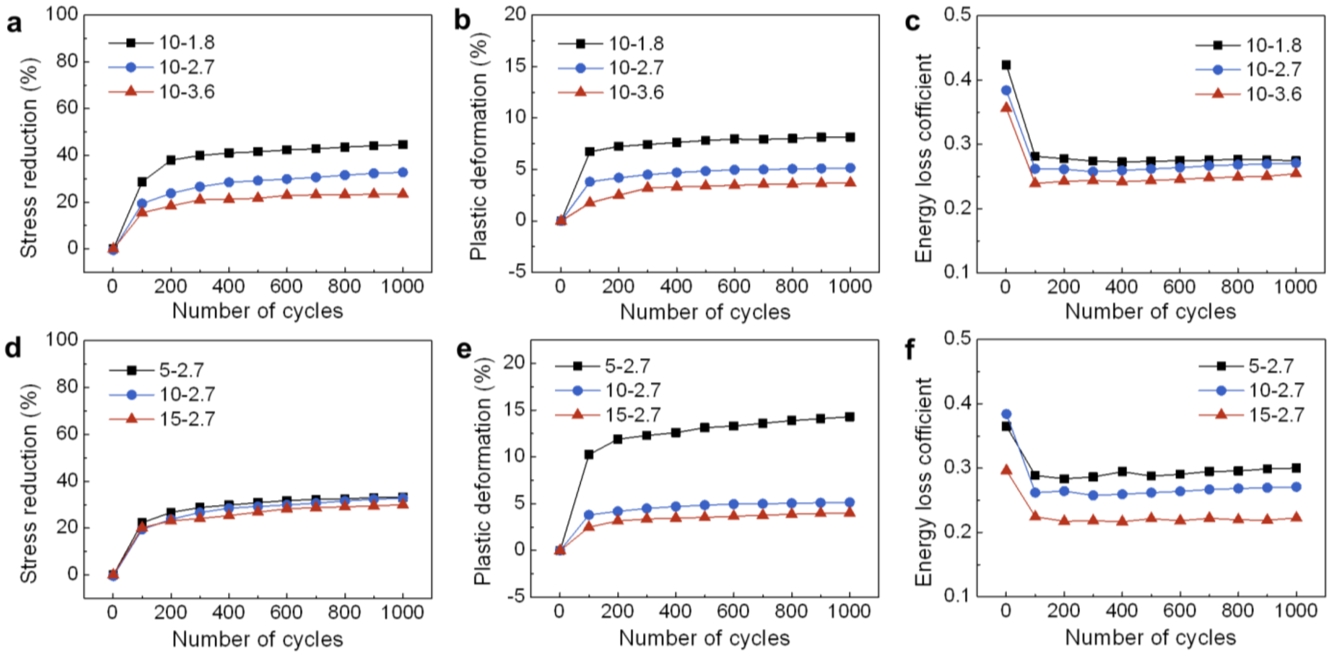

Supplementary Figure 21│Multicycle compression test results of the carbon monoliths . a-c, Change of maximum stress, plastic deformation and energy loss coefficient during the first 1000 compression cycles at the maximum strain of 80% for C-G monoliths made from different content of GO. **d-f,** Change of maximum stress, plastic deformation and energy loss coefficient during the first 1000 compression cycles at the maximum strain of 80% for C-G monoliths made from different content of CS. It is found that increasing the initial GO content would be beneficial to the structural stability of the ultimate C-G monoliths. On the one hand, graphene served as robust filler can enhance the mechanical property of the amorphous carbon skeleton. On the other hand, C-G monoliths made from more content of graphene have larger arch-shells (Supplementary Figs. 16), which should facilitate them to accommodate larger out-of-plan deformation. Furthermore, initial CS content also has a great influence to the structural stability of the ultimate C-G monolith. Lower content of CS would result in thin and defective lamellas of the C-G monolith (Supplementary Figs. 16, 17, and 19). At the same time, the shrinkage of the lamellas is insufficient, forming relatively flat arch-shells (Supplementary Figs. 16). Therefore, large plastic deformation happens at the first compression cycle due to the elimination of a lot of gaps between opposite arch structures (**e**). In the meantime, the defective lamellas with a lot of defects would dissipate much more energy in the compression cycle attributed to larger friction (**f**).

Supplementary Table 1 | Comparisons of mechanical performances of our carbon elastomer with relevant state-of-the-art low density compressible foams.

Materials	Density (mg cm^{-3})	Recovery Speed (mm s^{-1})	Energy loss coefficient (strain)	Cycles (strain)	Maximum Stress remaining	Plastic deformation	Ref
Ni Micro Lattices	14	-	0.8→0.42 (50%)	10 (50%)	~70%	3%	15
Carbon Nanotube Films	-	>2	0.64→0.47 (85%)	1000 (57%)	~76.8%	~7.5%	16
				1000 (85%)	~63%	14%	
CNT Arrays	-	2	0.58→0.49 (15%)	0.5×10^{6} (15%)	~53%	<5%	17
Graphene-Coated CNT Aerogel	14	11.2	0.33→0.3 (60%)	2000 (60%)	100	-	18
				10^{6}(6%)	100	-	
Graphene Cellular Monolith	5.1	117	0.83→0.67 (80%)	10 (80%)	~75%	-	4
				1000 (50%)	76%	7%	
3D Printed Graphene Aerogel	53	-	0.59→0.32 (50%)	10 (50%)	~38%	~15%	8
Graphene Spongy	1.1	-	-	1000 (90%)	~48%	-	19
				10^{6} (10%)	-	-	
Graphene Cellular Networks	6.1	-	0.85→0.55 (50%)	10 (50%)	~68%	5%	6
Electrospun Nanofibers Aerogels	9.6	24	0.55→0.4 (60%)	100 (60%)	~82%	4.8%	7
				1000 (60%)	75%	14.5%	
Tubular Graphene Form	9.8	-	0.72→0.62 (90%)	500 (80%)	~86%	<1%	20
				500 (90%)	~72%	<1%	
CNT Aerogel	5–10	>1.2	0.64→0.51 (50%)	100 (30%)	100%	8 %	21
				100 (60%)	100%	~17.5%	
Carbonaceous Nanofiber Aerogels	3.3	-	0.49→0.37 (60%)	100 (60%)	>80%	20.5%	22
Graphene Aerogels	3	-	0.75→0.62 (50%)	1000 (50%)	~67%	~3%	23
Graphene-CNT Aerogels	1	-	0.8→0.76 (50%)	1000 (50%)	88%	~10%	2
RF-Graphene Aerogels	>15.7	-	-	50% (50%)	~93%	11.4%	24
C-G Monolith (3.6-10)	13.8	580	0.2→0.19 (20%)	10^{6} (20%)	97%	0.6%	This work
			0.29→0.19 (50%)	2.5×10^{5} (50%)	86%	2%	
			0.36→0.26 (80%)	10^{3} (80%)	76%	3.6%	
			0.26→0.25 (90%)	10^{4} (80%)	60%	7%	

The third column shows energy loss coefficient in first cycle and after stabilized for the first several cycles.

Supplementary References

1. Yang, X., Tu, Y., Li, L., Shang, S. & Tao, X. M. Well-dispersed chitosan/graphene oxide nanocomposites. *ACS Appl. Mater. Inter.* **2**, 1707-1713 (2010).
2. Sun, H., Xu, Z. & Gao, C. Multifunctional, ultra-flyweight, synergistically assembled carbon aerogels. *Adv. Mater.* **25**, 2554-2560 (2013).
3. Qian, Y. Q., Ismail, I. M. & Stein, A. Ultralight, high-surface-area, multifunctional graphene-based aerogels from self-assembly of graphene oxide and resol. *Carbon* **68**, 221-231 (2014).
4. Qiu, L., Liu, J. Z., Chang, S. L., Wu, Y. & Li, D. Biomimetic superelastic graphene-based cellular monoliths. *Nat. Commun.* **3**, 1241 (2012).
5. Zou, J. H. *et al.* Ultralight Multiwalled Carbon Nanotube Aerogel. *ACS Nano* **4**, 7293-7302 (2010).
6. Barg, S. *et al.* Mesoscale assembly of chemically modified graphene into complex cellular networks. *Nat. Commun.* **5**, 4328 (2014).
7. Si, Y., Yu, J., Tang, X., Ge, J. & Ding, B. Ultralight nanofibre-assembled cellular aerogels with superelasticity and multifunctionality. *Nat. Commun.* **5**, 5802 (2014).
8. Zhu, C. *et al.* Highly compressible 3D periodic graphene aerogel microlattices. *Nat. Commun.* **6**, 6962 (2015).
9. Worsley, M. A., Kucheyev, S. O., Satcher, J. H., Hamza, A. V. & Baumann, T. F. Mechanically robust and electrically conductive carbon nanotube foams. *Appl. Phys. Lett.* **94**, 073115 (2009).
10. Tang, Z., Shen, S., Zhuang, J. & Wang, X. Noble-metal-promoted three-dimensional macroassembly of single-layered graphene oxide. *Angew. Chem. Int. Ed.* **49**, 4603-4607 (2010).
11. Chen, W., Li, S., Chen, C. & Yan, L. Self-assembly and embedding of nanoparticles by in situ reduced graphene for preparation of a 3D graphene/nanoparticle aerogel. *Adv. Mater.* **23**, 5679-5683 (2011).
12. Sui, Z. Y., Meng, Q. H., Zhang, X. T., Ma, R. & Cao, B. Green synthesis of carbon nanotube-graphene hybrid aerogels and their use as versatile agents for water purification. *J. Mater. Chem.* **22**, 8767-8771 (2012).
13. Zhang, X., Liu, J., Xu, B., Su, Y. & Luo, Y. Ultralight conducting polymer/carbon nanotube composite aerogels. *Carbon* **49**, 1884-1893 (2011).
14. Zhang, X. T. *et al.* Mechanically strong and highly conductive graphene aerogel and its use as electrodes for electrochemical power sources. *J. Mater. Chem.* **21**, 6494-6497 (2011).
15. Schaedler, T. A. *et al.* Ultralight metallic microlattices. *Science* **334**, 962-965 (2011).
16. Cao, A., Dickrell, P. L., Sawyer, W. G., Ghasemi-Nejhad, M. N. & Ajayan, P. M. Super-compressible foamlike carbon nanotube films. *Science* **310**, 1307-1310 (2005).
17. Suhr, J. *et al.* Fatigue resistance of aligned carbon nanotube arrays under cyclic compression. *Nat. Nanotechnol.* **2**, 417-421 (2007).
18. Kim, K. H., Oh, Y. & Islam, M. F. Graphene coating makes carbon nanotube aerogels superelastic and resistant to fatigue. *Nat. Nanotechnol.* **7**, 562-566 (2012).
19. Wu, Y. *et al.* Three-dimensionally bonded spongy graphene material with super compressive elasticity and near-zero Poisson's ratio. *Nat. Commun.* **6**, 6141 (2015).
20. Bi, H., Chen, I. W., Lin, T. & Huang, F. A New Tubular Graphene Form of a Tetrahedrally Connected Cellular Structure. *Adv. Mater.* **27**, 5943-5949 (2015).
21. Gui, X. *et al.* Carbon nanotube sponges. *Adv. Mater.* **22**, 617-621 (2010).
22. Liang, H. W. *et al.* Macroscopic-scale template synthesis of robust carbonaceous nanofiber hydrogels and aerogels and their applications. *Angew. Chem. Int. Ed.* **51**, 5101-5105 (2012).
23. Hu, H., Zhao, Z., Wan, W., Gogotsi, Y. & Qiu, J. Ultralight and highly compressible graphene

aerogels. *Adv. Mater.* **25**, 2219-2223 (2013).

24. Wang, X. *et al.* Scalable Template Synthesis of Resorcinol-Formaldehyde/Graphene Oxide Composite Aerogels with Tunable Densities and Mechanical Properties. *Angew. Chem. Int. Edit.* **54**, 2397-2401 (2015).
